# *In situ* high-resolution structure of the baseplate antenna complex in *Chlorobaculum tepidum*

**DOI:** 10.1038/ncomms12454

**Published:** 2016-08-18

**Authors:** Jakob Toudahl Nielsen, Natalia V. Kulminskaya, Morten Bjerring, Juha M. Linnanto, Margus Rätsep, Marie Østergaard Pedersen, Petar H. Lambrev, Márta Dorogi, Győző Garab, Karen Thomsen, Caroline Jegerschöld, Niels-Ulrik Frigaard, Martin Lindahl, Niels Chr. Nielsen

**Affiliations:** 1 Center for Insoluble Protein Structures (inSPIN), Aarhus C DK-8000, Denmark; 2 Interdisciplinary Nanoscience Center (iNANO), Aarhus University, Gustav Wieds Vej 14, Aarhus C DK-8000, Denmark; 3 Department of Chemistry, Aarhus C DK-8000, Denmark; 4 Institute of Physics, University of Tartu, W. Ostwaldi Street 1, Tartu 50411, Estonia; 5 Novo Nordisk a/s, Novo Nordisk Park, Måløv 2760, Denmark; 6 Hungarian Academy of Sciences, Biological Research Centre, Temesvári krt. 62, Szeged 6726, Hungary; 7 Karolinska Institutet, Department of Biosciences and Medical Nutrition, Hälsovägen 7, Huddinge 141 83, Sweden; 8 Section for Marine Biology, Department of Biology, University of Copenhagen, Strandpromenaden 5, Helsingør DK-3000, Denmark

## Abstract

Photosynthetic antenna systems enable organisms harvesting light and transfer the energy to the photosynthetic reaction centre, where the conversion to chemical energy takes place. One of the most complex antenna systems, the chlorosome, found in the photosynthetic green sulfur bacterium *Chlorobaculum (Cba.) tepidum* contains a baseplate, which is a scaffolding super-structure, formed by the protein CsmA and bacteriochlorophyll *a*. Here we present the first high-resolution structure of the CsmA baseplate using intact fully functional, light-harvesting organelles from *Cba. tepidum*, following a hybrid approach combining five complementary methods: solid-state NMR spectroscopy, cryo-electron microscopy, isotropic and anisotropic circular dichroism and linear dichroism. The structure calculation was facilitated through development of new software, GASyCS for efficient geometry optimization of highly symmetric oligomeric structures. We show that the baseplate is composed of rods of repeated dimers of the strongly amphipathic CsmA with pigments sandwiched within the dimer at the hydrophobic side of the helix.

## Abstract (web summary)

The chlorosome of the photosynthetic bacterium *C. tepidum* harvests light and transfers the energy to the photosynthetic reaction centre. Here the authors determine the structure of the baseplate, a scaffolding super-structure, to show that the baseplate consists of rods of repeated CsmA dimers containing pigment molecules.

## Graphical abstract

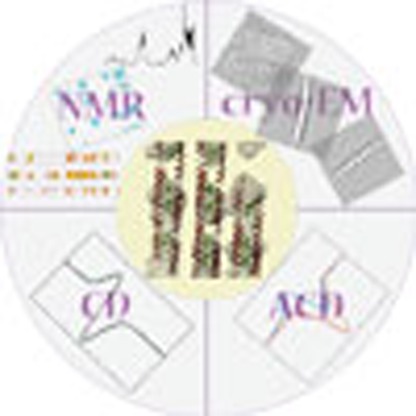

Photosynthetic organisms use a variety of structurally and evolutionary unrelated light-harvesting antenna complexes^1^. These antennas are essential for the organisms to harvest light energy, which is transferred to the photosynthetic reaction centre and converted to chemical energy. Most photosynthetic antennas consist of a protein scaffold upon which pigment molecules are organized. The only exception is the chlorosome organelle of green bacteria^2^, where bacteriochlorophyll (BChl) *c*, *d*, or *e* molecules self-assemble into protein-free, rod-like nanotubes^3^,^4^. Detailed structural information is available for representatives of many light-harvesting antenna systems^3^,^5^,^6^,^7^,^8^,^9^,^10^,^11^, but not for the proteinaceous baseplate of chlorosomes^2^. This structure has three essential functions: (i) ensuring the structural integrity of the chlorosome organelle, (ii) connecting the chlorosomes to the photosynthetic reaction centre in the cell membrane and (iii) transferring excitation energy from the protein-free BChl *c/d/e* antennae towards the reaction centre.

Previous studies have shown that roughly 1,000–2,000 of CsmA protein subunits, each binding one BChl *a* pigment molecule, constitute the baseplate^12^. In addition, it has been seen by freeze-fracture electron microscopy (EM) that the resulting structure appears as a two-dimensional (2D) crystal-like structure^13^,^14^. The baseplate is oriented towards the cytoplasmic membrane and in the *Cba. tepidum* attaches to the so-called Fenna-Matthews-Olson (FMO) protein complex, which is the last antenna before the energy reaches the reaction centre (Fig. 1a,b)^2^.

Structural characterization of the CsmA baseplate is very challenging, due to the complex and heterogeneous composition of the native molecular assembly that precludes analysis with traditional techniques such as X-ray crystallography or liquid-state nuclear magnetic resonance (NMR). The baseplate structure is unlike any other light-harvesting antenna in that it appears impossible to isolate or otherwise produce a small defined intact CsmA oligomer from the baseplate in a stable soluble form suitable for studies of the *in vivo* structure, in particular, because it disintegrates irreversibly upon detergent treatment^15^,^16^ and because *in vitro* reconstitution of CsmA and BChl *a* cannot reconstitute the baseplate structure^17^.

The novel approach in the present work to structural studies of the baseplate became possible with a *bchK* mutant of *Cba. tepidum* that forms modified chlorosomes, termed carotenosomes, with a fully functional baseplate formed by CsmA protein and BChl *a* pigment but lacking self-assembled BChl *c* antenna elements^15^. Removing the vast excess of BChl *c* molecules (100,000–200,000 per chlorosome) unmasked the baseplate allowing direct studies of the baseplate *in vivo*. Despite the simplicity of the carotenosomes, the functionality of the organelles; however, remains intact as the bacteria are able to grow photosynthetically using BChl *a* and carotenoids as the sole antenna pigments. It should be noted that while BChl *c* is nonessential for the survival of *Cba. tepidum*, removing CsmA and thus the baseplate cannot give viable mutants^18^. Initially, we presented a liquid-state NMR structure of CsmA in organic solution^19^ and later extended the investigation with solid-state NMR resonance (ssNMR) assignments and secondary structure information^20^ for the CsmA peptides residing *in situ* in the baseplate of the carotenosome^15^.

Finally, here we conclude the structural characterization of the CsmA baseplate by presenting the high-resolution structure of the CsmA carotenosome baseplate. ssNMR data provide structural information in terms of distances and dihedral angle constraints. However, due to the local nature of these constraints, it was not possible to calculate the structure of the baseplate using these constraints alone. Therefore, the ssNMR data was complemented with a 3D density model derived using cryo-electron microscopy (cryo-EM) providing constraints for the global structure of the baseplate. Due to considerable variation in the quaternary structure of the carotenosomes (different lengths and curvatures, see below) it was only possible to derive a three-dimensional (3D) density model of medium-low resolution. Previously, ssNMR and microscopy data have been combined for high-resolution structure determination of supramolecular assemblies^21^,^22^,^23^,^24^. Furthermore, the unique composition of chlorosome complexes containing aggregates of bacteriochlorophyll molecules provide unique opportunities for near infrared spectroscopy, which is highly sensitive to ligand conformations, for comparing experimental data with structure-based *ab initio* calculated spectra^25^,^26^,^27^. Here we derive structure constraints and validation of the structure in terms of orientation between BChl *a* pigments. We compare the observed and *ab initio* structure using back-calculated data from absorption spectra, linear dichroism (LD) and circular dichroism (CD) in both isotropic and anisotropic phase (ACD, oriented samples).

Structure calculation of highly symmetric protein aggregates represents a great challenge. Although rotational and translational symmetries can be modelled in standard software packages, such as Xplor-NIH, using a symmetry potential to equal pairs of distances^28^, this is in practice problematic, due to issues with ineffective conformational sampling caused by high-energy barriers between different symmetric conformations. The problem can be addressed by implicit modelling of symmetry-related molecules and possibly explicit variation of parameters describing the symmetry as implemented for more simple and specific symmetries^29^,^30^,^31^,^32^. However, we did not find software suitable for the specific symmetry of the CsmA baseplate, and therefore we developed new software, GASyCS, for calculating the structure of highly symmetric oligomeric structures. GASyCS uses a genetic algorithm framework^33^,^34^ to search global conformational space and optimizes the structure following a basin hopping strategy^35^ (Supplementary Methods).

## Results

### Characterization of the overall structure of the carotenosome

**Figure 1:**
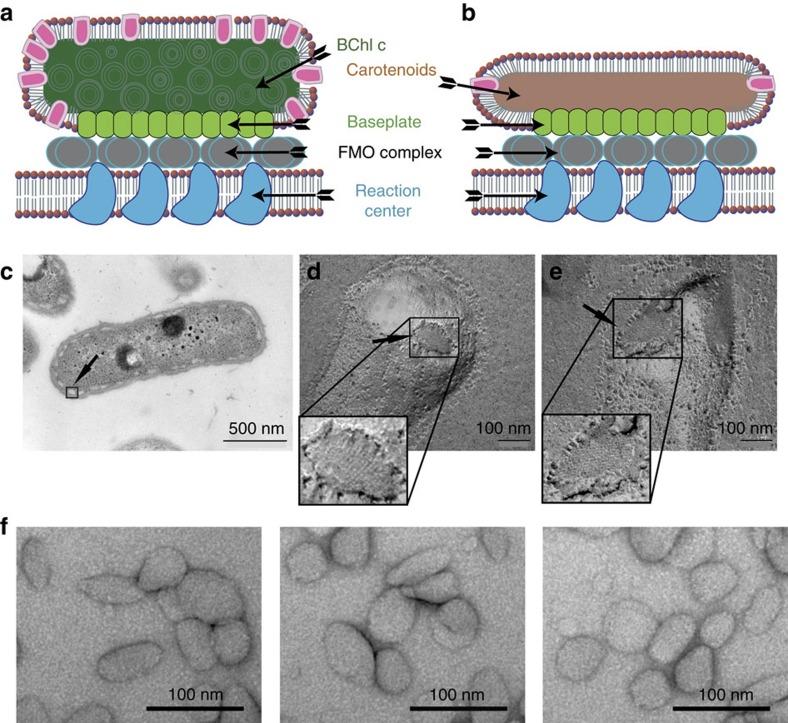
Organization and structure overview of the chlorosome and the carotenosome. (**a**) Cartoon of a chlorosome. (**b**) Cartoon of a carotenosome showing CsmA (green), non-CsmA polypeptides (magenta), the surrounding lipid monolayer (red), BChl *a* rods (dark green), carotenoids (brown), the FMO complex (grey) and the reaction centres (dark blue) in the cytoplasmic membrane. (**c**) Plastic (epon) embedded thin section of the whole bacterium *Cba. tepidum*, arrow points to a boxed chlorosome. Baseplates of freeze fractured *Cba. tepidum* cells on a (**d**) wild-type chlorosome, (**e**) *bchK* mutant, that is, a carotenosome (zoom shows higher resolution part of the images). (**f**) Isolated carotenosomes prepared by negative stain embedding with uranyl acetate (Supplementary Note 1 and Supplementary Figs 1 and 2).

Chlorosomes, located at the intracellular side of the cytoplasmic membrane in *Cba. tepidum*, appear as small oval signatures as seen in a thin section image (Fig. 1c). Freeze-fracture imaging of *Cba. tepidum* bacteria reveals chlorosomes with a noticeably repeating striated signature, which earlier was also observed for *Chloroflexus (Cfx.) aurantiacus* and *Chlorobium limicola*^13^,^14^. The periodic flat structure represents the antenna baseplate system, which has been in focus of the present study (Fig. 1d and Supplementary Figs 1 and 2). As illustrated schematically in Fig. 1a, the baseplate is a part of the chlorosome, containing a varity of components, including lipids, proteins, carotenoids and other pigment molecules. The corresponding organization of the reduced number of components in the variant chlorosome, called the carotenosome, is visualized in Fig. 1b. To clarify, whether the baseplate remains intact after the bacterial mutation, freeze-fracture EM was used for careful examination of both specimens. The striated area, depicted for the original (non-mutated) chlorosomes (Fig. 1d) and for a *bchK* mutant cell (Fig. 1e) establishes the similarity between the chlorosome and carotenosome baseplates. Indeed, this would be the expected situation in terms of organelle functionality, since the function of the carotenosome is independent of the presence of BChl *c* and dependent on BChl *a* molecules.

Further, isolated carotenosomes have been studied by transmission EM using both negatively stained and ice-embedded samples. The sample quality in conjunction with the overall shape and size of the species was addressed by negative-stain microscopy, while the structural information of the long-range organization of the CsmA protein by cryo-EM. The carotenosomes studied by negative stain EM, reveal shape variations ranging between 60–80 nm in length and 40–80 nm widths, some with more round shape and some more elongated, but generally smaller and more irregular than the chlorosomes as seen by negative stain EM and other transmission electron microscopy and atomic force microscopy techniques reported earlier^15^,^36^ (Fig. 1f and Supplementary Note 1).

### Generation of a 3D density model for the baseplate structure

**Figure 2:**
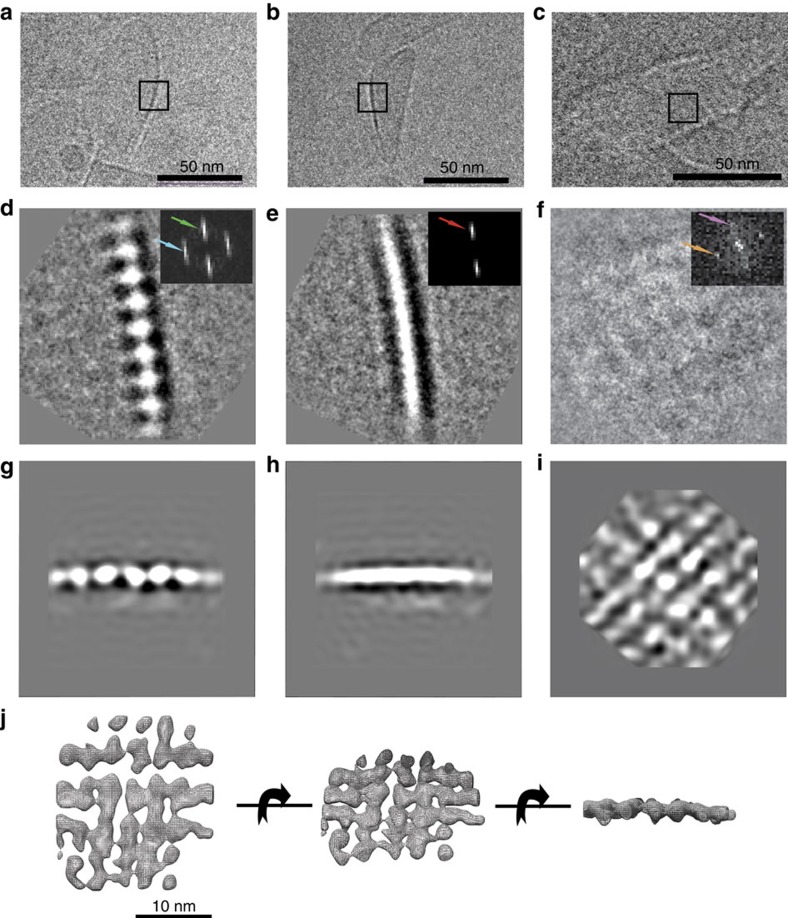
Carotenosome structure organization by cryo-EM. (**a**–**c**) Cryo-EM examples of ice-embedded carotenosomes that contains characteristic features of the baseplate. (**d**–**f**) 2D class-averages from the three different views taken to represent two side views and one top view, all 90° apart: (**d**) string of ‘beads' (**e**) single stripe view, (**f**) weak-contrast mesh-like view (box size is 30.7 nm). Insets: Power spectra indicating repeat directions and distances in the class-averages. Green arrow corresponds to 47 Å and represents the thickness of the baseplate, whereas the blue arrow corresponds to 33 Å, indicating a specific internal repeating distance in one direction and red arrow revealing a repeating distance of 41 Å in another direction. Orange and purple arrows reveal repeating distances of 33.8 and 35.7 Å respectively. (**g**–**i**) Three perpendicular projections of the refined 3D model. (**j**) Electron density model showing the density map as a mesh wireframe (Supplementary Fig. 8b).

Cryo-EM data of the ice-embedded isolated carotenosomes is, on one hand, in agreement with negative stain data and, on the other hand, reveals more detailed molecular complex structure. Captured images clearly show supramolecular organization of the protein rows, originating from the baseplate (Fig. 2a–c). From 270 images, 1790 individual baseplate views from different orientations were manually selected using the e2boxer.py in EMAN2 software package^37^ and analysed using single particle analysis. We observed three most characteristic signatures in the cryo-EM pictures (Fig. 2d–f): a line of equidistant ‘beads', a single stripe and a weak-contrast mesh-like pattern. Further, chosen classes were subjected to distance analysis, using power spectra (Fig. 2d–f, insets). Repeat distances for the beady view from different class-averages were very well conserved at 33 Å (which is consistent with the previously reported results^38^,^39^). The thickness of the baseplate, which can be defined from the single stripe and beady views, shows variation in a range of 41–47 Å (Fig. 2a,b,d,e), depending on the view angle. Here, the estimation for a lattice constant was 33 Å. A top view of the baseplate revealed repeating distances of 33.8 and 35.7 Å with an 81° angle to each other, which at closer examination discloses the checkered 2D pattern consistent with the top view of the refined structure (Fig. 2f).

An initial 3D electron density model for the baseplate (Fig. 2j) was generated revealing the baseplate as an array of aligned rod-like structures with ca. 33–35 Å spacing (compared to the 33 Å in the class-averages) and a thickness of 41–47 Å (which is also consistent with the class-averages data). After the calculation of the initial model, the reconstruction was refined using the set of 1790 individual particles aligned first in Sparx^40^ software and subsequently in Euler angle space using Strul^41^. A new 3D model was calculated using weighted back-projection in Spider^42^ (Fig. 2g–i). The final reconstruction density map is presented in Fig. 2j. The resolution estimate is 19.1 Å (FSC=0.143 criteria, Supplementary Fig. 3b and Supplementary Methods).

### Structural constraints from ssNMR

**Figure 3:**
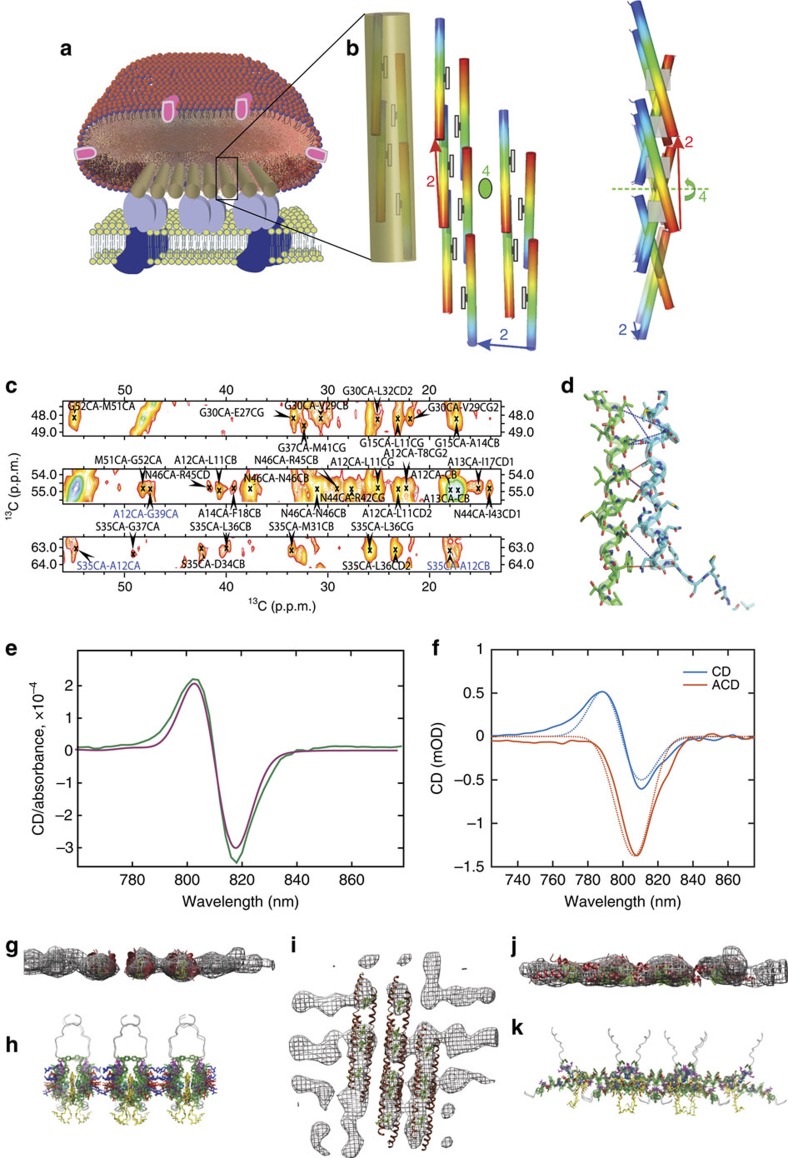
Structure calculation data and validation. (**a**) Cartoon of the carotenosomes. (**b**) Final structure of the baseplate shown as a 3D cartoon highlighting the degrees of freedom for the symmetry with numbers (Supplementary Note 2) showing CsmA helices with rainbow colours indicating N- and C-terminal in blue and red, respectively, with added drawings of the BChl *a* ligands (coordinated to H25) as grey boxes. The cluster of monomers covered by the transparent cylinder constitutes the rods in the cryo-EM density model. (**c**) Representative strip plot from a solid-state NMR DARR (200 ms mixing time) spectrum with peak assignment annotated highlighting inter-chain constraints in blue and intra chain in black, the average signal-to-noise ratio for the three highlighted peaks assigned to inter-chain contacts was estimated to 4.1. (**d**) Molecular representation of the interface between two CsmA monomers showing inter-chain constraints as blue dotted lines and possible long-range constraints in red. (**e**) Overlay of experimental (green) and calculated (magenta) isotropic CD spectra of the carotenosomes from *Cba. tepidum* in baseplate absorption region at 77 K. Corresponding spectrum for the Qy region is shown in Supplementary Fig. 9. (**f**) Overlay experimental (solid lines) and calculated (dotted lines) of isotropic CD and anisotropic CD (ACD) spectra in the near infrared region acquired at room-temperature (data from Supplementary Fig. 5), (**g**,**i**,**j**) Cryo-EM density model as a mesh overlaid BChl *a*-CsmA structures derived from solid-state NMR and visualized by Chimera^67^, Red: the CsmA protein chain, green: BChl *a*, note that the threshold for visualizing the density is set relatively high to increase readability of the figure. (**h**,**k**) The baseplate structures from the corresponding angles are shown as cartoon diagrams below the meshes (**g**,**j**). Yellow: BChl *a*, Green: aromatic, Red: negatively charged and Blue: positively charged residues (see also Fig. 4).

From our previous solid-state NMR investigations, we could, based on secondary chemical shift data, conclude that the CsmA monomers *in situ* in the baseplate display a largely α-helical structure encapsulated into a fully symmetric baseplate super-structure^20^. Furthermore, we conclude now, based on analysis of H25 side chain chemical shifts (Supplementary Note 2) that BChl *a* ligands are coordinated 1:1 via H25. CD spectra analysis and *ab initio* calculations (Supplementary Note 2) demonstrate that the basic building block of the baseplate structure is a symmetric CsmA dimer, which is translated along two perpendicular directions to form a two-dimensional lattice structure (Fig. 3b).

To provide detailed information about the structure, a solid-state NMR DARR ^13^C–^13^C 2D spectrum with a long (200 ms) mixing time was acquired (see Methods). Cross peaks in the spectrum are signatures of close proximity between two carbons assigned to the corresponding chemical shifts. Cross peaks with chemical shift overlapping with the carotenoid and/or lipid ranges were not used. But nonetheless, due to unique chemical shift regions for certain protein signals, it was possible to exploit resonances corresponding to C′ and Cα for all residues and, in addition, Cβ from T and S residues, which appear in the spectrum separately from the carotenoids signals (Supplementary Fig. 4a). For each cross peak all assignment possibilities consistent with the assigned chemical shifts within a window of 1.35 p.p.m. around the peak position were considered (that is, with maximum distance of 0.675 p.p.m., comparable to typically observed line widths of ca. 0.8 p.p.m. measured as half width at half height). Representative excerpts of the DARR spectrum is shown in Fig. 3c with the most likely assignment indicated for each peak. All constraints were implemented as ambiguous distance constraints corresponding to finding the conformation that optimizes the sum of contributions to the cross peak intensity from all considered carbon pairs. This implementation accounts for both all intra-chain contacts but also for all the possible inter-chain contacts for the corresponding carbon atom pair. A total of 60 non-trivial distance constrains were used for the calculation and, in addition, 88 dihedral angle restraints derived using TALOS+(ref. 43) from the assigned chemical shifts were used to restrain the backbone conformation.

### Constraints on the pigment orientation from LD, CD and ACD

Low temperature CD spectra (Fig. 3e) were acquired for the carotenosomes in the near infrared region, since the absorbance and corresponding CD signal in that region is highly sensitive both to the local geometry and the overall structure of the baseplate. Further constraints were derived by measuring linear dichroism (LD) and anisotropic CD (ACD) spectra from macroscopically aligned carotenosomes at room temperature (Fig. 3f). The LD and ACD spectra constrain the orientation angles of the transition dipole moments contributing to the absorption and CD bands, respectively (Supplementary Note 3 and Supplementary Fig. 5, which also includes spectra for the visible light region).

### Calculation of the CsmA-BChl *a* baseplate structure

The structure of the CsmA-BChl *a* baseplate was calculated in four phases, using a combination of all the available experimental data, in each phase calculating a number of structure models for use in the next phase: (i) monomer structure calculation (ii) global structure search (iii) candidate filtering and (iv) structure refinement. In the first phase candidate monomer structures were calculated based on the TALOS+ derived backbone angle constraints. These monomer structures were used as starting points for the second phase where the monomer structures were rotated and translated in a configuration, which preserves the full symmetry, that is, in the symmetry constraints space (Supplementary Information and Methods). The structure candidates having the lowest energy were further refined using Xplor-NIH and distance similarity constraints together with non-strict crystallographic symmetry^44^ to enforce symmetric conformation applying to both the ambiguous distance constraints and the dihedral angle constraints derived using TALOS+ producing a large set of different candidate baseplate structure models. In the third phase, as a filtering step, CD spectra were back-calculated using quantum chemical methods^45^,^46^ based on the above candidate structural models (see Methods and Supplementary Methods) and compared with the observed spectra rejecting candidates models for which the spectra did not match (see below and Methods). The cryo-EM density was used to further reject a subset of the models inconsistent with the cryo-EM density model (see example of rejected models and the corresponding force field energies in Supplementary Figs 6 and 7 and Supplementary Fig. 3a). In the fourth and final phase of the structure determination, the cryo-EM density data was included in the NMR structure calculation protocol to refine the final structure (Supplementary Methods and Methods). Finally, to validate the final carotenosome baseplate structure, isotropic low temperature CD and anisotropic CD spectra were back-calculated revealing a close agreement with the observed spectra (Fig. 3e,f). Hence, by combining all types of experimental constraints: local high-resolution structural information from solid-state NMR, orientation between BChl *a* pigments from isotropic and anisotropic CD, LD, *ab initio* calculations, and global structural information from cryo-EM, together with the symmetry information, it was possible to derive a unique and precise structure for the CsmA-BChl a baseplate.

**Figure 4:**
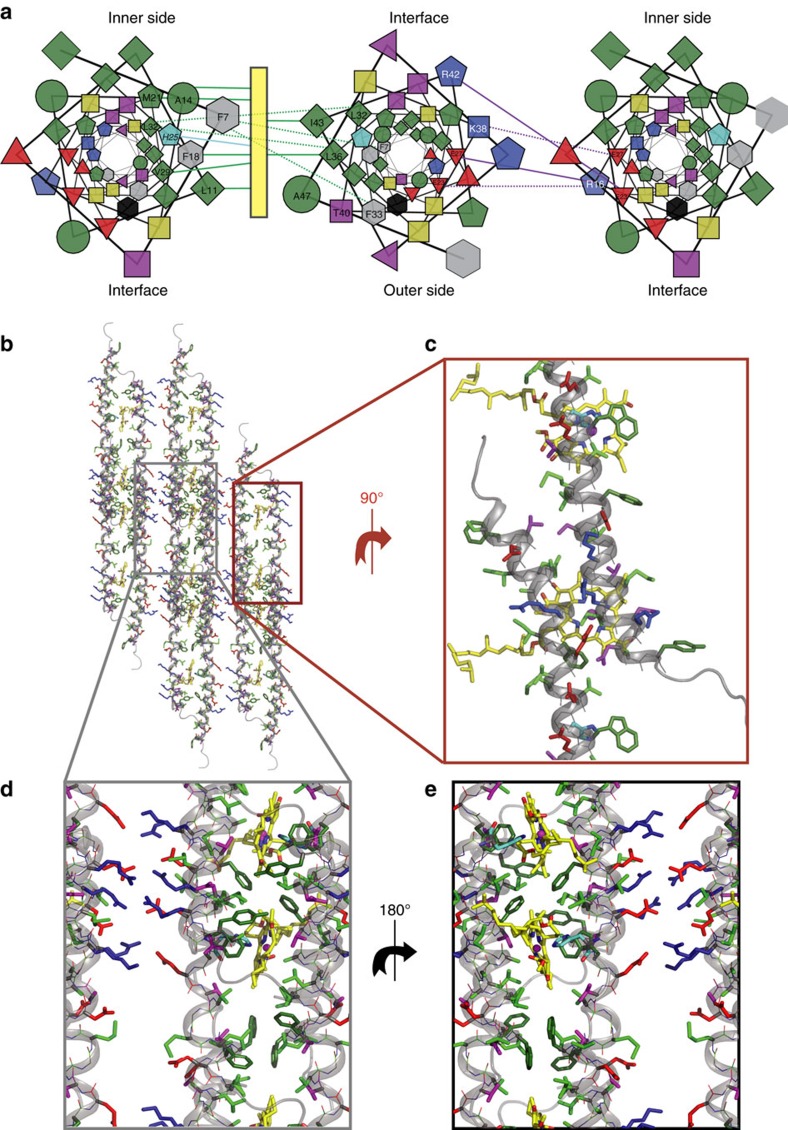
The CsmA baseplate structure. (**a**) Helical wheel projections depicting three neighbouring helices viewed down the helix axis (colour code; green: hydrophobic, grey: aromatic, black: W, cyan: H, yellow: G, red: negatively charged, blue: positively charged). The symbols represent different residue types: G (circle), S/T/G/K (square), I/L/V (diamond), F/Y/W (hexagon), H/M/R (pentagon), and D, E, and Q/N (triangle pointing up, down and left, respectively). Purple lines indicate electrostatic interactions between oppositely charged residues, green lines highlight residues closely located to the BChl *a* pigment (yellow rectangle), while dotted lines represent hydrophobic packing between amino acids. The residues present on outer and inner side are annotated as well as the interface between two helices related by translational symmetry. (**b**–**e**) Molecular representation of the baseplate showing CsmA with cartoon and lines and side chains with sticks for the central residues 6–48. BChl *a* are shown as yellow sticks with N and O coloured in blue and red, respectively, hydrogen atoms are omitted for clarity. The colour code is the same in (**a**) except G is green and aromatic residues are shown with a darker green. (**b**) top view, (**c**) zoom on the translation interface, (**c**–**e**) zoom on the two interfaces viewed from the outer and inner side (the phytyl chains show here) of the carotenosome, respectively. See also Fig. 5.

The final structure shown in Fig. 4 has the lowest total energy and fitted all experimental data very well and significantly better than all the other competing models (Supplementary Fig. 6 and 7 and Supplementary Fig. 3a) revealing a good agreement with all solid-state NMR constraints, cryo-EM density (judged by the overlay with the density model in Fig. 3g,i,j), isotropic CD (Fig. 3e), anisotropic CD spectra (Fig. 3f) and LD spectra (Supplementary Fig. 5) (see Methods, Supplementary Methods and Supplementary Note 4). Some of the inter-chain distance constraints found for the CsmA baseplate structure are visualized in Fig. 3d defining the local structure with high precision. The global organization of the baseplate structure is also well determined through the relative orientation between BChl *a* pigments, which can be deduced from the CD spectrum analysis (see Methods) and through agreement with the cryo-EM density model. Restraint and structure statistics is provided in Table 1.

### Description of the baseplate structure

**Figure 5:**
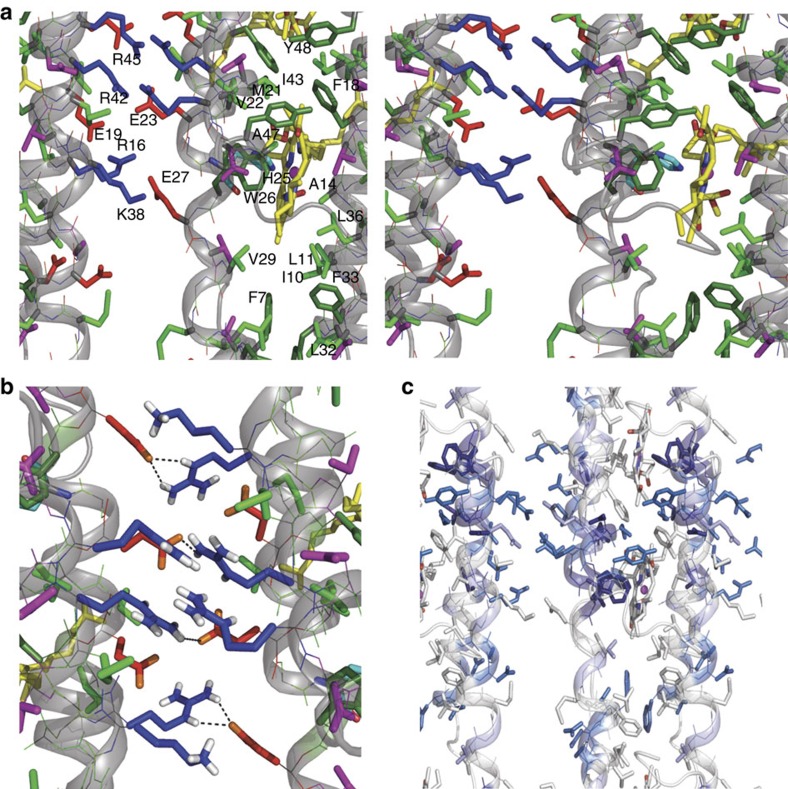
Details of the structure of the CsmA baseplate. (**a**) Stereo view of details in the baseplate structure, see legend to Fig. 4. (**b**) Side chain hydrogen bonding in CsmA baseplate structure. H atoms are shown for polar side groups, O atoms are shown in orange. Hydrogen bonds are highlighted with black dashed lines. (**c**) Molecular structure of the CsmA baseplate visualizing homologous residues; fully conserved residues: dark blue, strongly similar residues: light blue, weekly similar: pale blue. The aligned species and corresponding sequences are shown in Supplementary Table 2.

In accord with indications from earlier studies, the CsmA monomers form helical structures with a slight curvature^20^. The data presented here reveal that the helices form rods by head-to-tail translational association in a tight interface allowed by the small amino acids of in particular G15, G39 and A12 flanked by other relatively small polar amino acids such as T8, S35 and N46 (Figs 3d and 4c). The helices are amphipathic (Fig. 4a) leading to formation of a hydrophobic and a hydrophilic interface (Fig. 4b,d,e). The hydrophilic interface pairs the side of the helices with electrostatic interactions through oppositely charged side-chains (Fig. 4c). The hydrophobic interface is further stabilized by symmetry-related packing of aromatic rings of F7/F7* and F18/F18* and clustering of other hydrophobic residues (Figs 4d,e and 5a). In particular, hydrogen bonds between side chains are formed for R42/E23 and R16/E27 and salt-bridges for R16/E23 and K38/E27 (Fig. 5b) and within the same rod for R45/E23 and R42/E19. The putative salt-bridges are supported by narrow lines in the solid-state NMR spectra for the side-chain C′ for these negatively charged residues. Since the helixes are tilted slightly relative to baseplate plane, the axis of rotation parallel to the baseplate normal leads to a small angle (ca. 25°) criss-cross pattern of the helices (Fig. 3b,k), as also observed in another helical oligomeric structure^47^. Due to this symmetry, the only inter-chain contacts are between residues close in sequence (for example, L32 and F33*) and a homo-interface pairing, packing the BChl *a* ligand coordinated to H25 tightly with hydrophobic side-chains such as L11, A14, F18, M21, W26, V29, L36 and I43 (Fig. 4a).

The BChl *a* ligand coordinates to the side-chain of H25 and form pairs, which are almost co-planar orthogonal to the plane of the baseplate and with the centres separated by ca. 15 Å along the rod axis. This geometry is consistent with the LD spectrum, the isotropic and the anisotropic CD spectra (Fig. 3e,f and Supplementary Fig. 5). In contrast, different orientations or a closer contact would lead to a totally different CD spectrum with additional CD bands, different rotation strengths values or mirror image CD shapes as compared with the spectrum, shown in Fig. 3e (Supplementary Note 2 for further details).

The symmetry of the baseplate defines two different sides of the baseplate. One, believed to face the inner side of the carotenosome (Fig. 4a,c), contains the phytyl tails of the BChl *a* ligands together with the N-terminal part and one side of the helix. This inner side is decorated by hydrophobic side chains, which might form anchoring points for carotenoid molecules and the tails of the esterifying alcohols of BChl *c* in the wild-type chlorosome. The other outer side consists of the C-terminal part of the helix as shown before^48^ and another side of the helix containing three aromatic residues, W26, F33 and Y48 and some hydrophilic side chains, which might constitute the binding site for the FMO complex^49^,^50^.

## Discussion

In studies on chlorosomes by cryo-EM, striations parallel to the long axis of the chlorosomes were observed and assigned to rods or lamellae formed by BChl *c* (refs 3, 39, 51, 52). Interestingly, perpendicular striations and other features (as in Fig. 1d,e) that could be assigned to the baseplate were also detected in both *Cfx. aurantiacus* and *Cba. tepidum* chlorosomes^3^,^39^,^51^,^52^. For the latter organism, work by Oostergetel *et al.*^51^, has shown a repeat distance of 33 Å from rows of protein similar to our beady view (Fig. 2a) as well as a from a side view and with an angle of 40° with the long axis of the chlorosome. In the carotenosomes lacking BChl *c* rods with such an angle cannot be formed.

Compared with the *Cba. tepidum* chlorosome with a thickness of 350–600 Å (refs 15, 39), chlorosomes from *Cfx. aurantiacus,* are thinner with 100–200 Å (ref. 39), hence baseplates can be clearly seen^39^,^52^ in particular in the high-light grown^27^ organelles. In agreement with our observations, it was argued that the baseplate building block is ca. 33 Å in diameter^39^.

For the carotenosomes an approximate thickness of the baseplate could be obtained (Fig. 2d,e), a measurement obscured by the BChl *c* interior in the chlorosomes. The baseplate thickness of 44±3 Å (obtained from 14 distance measurements in EM power-spectra (Supplementary Fig. 8) compares well with the thickness of the entire carotenosome, measured by atomic force microscopy to 43±8 Å (ref. 15). Altogether, cryo-EM results point to a baseplate structure conserved between at least these two species with a lattice of 33 Å in both directions in the plane and about 44 Å thick.

Earlier studies combining ssNMR constraints and microscopy-derived density models^21^,^22^,^23^,^24^ profited on a larger number of distance constraints and a density model with a higher resolution. However, we stress out here that the present study is performed in the context of the full carotenosomes where both lipids and carotenoids are abundant in the native environment in which some variations is seen for the quaternary structure of the carotenosomes. As a natural result, this true variation is reflected in the precision of the experimental data, but on the other hand, the accuracy of the data is not biased by purification or other non-native manipulations of the baseplate system. The carotenosomes are prone to stick to each other and to take preferred orientations complicating the reconstruction process and the resulting resolution. To possibly get better cryo-EM and ssNMR data, one could remove carotenoids and quinones not necessary for an intact baseplate. The carotenosomes may contain loosely bound carotenoids that would allow removal by a hexane wash similar to hexane washing of chlorosomes from *Cba. tepidum*^53^,^54^. An alternative procedure (work in progress) could be to genetically eliminate all carotenoids, because carotenoids appear not to be essential for the structural integrity of the baseplate structure^55^. Another approach to possibly obtain better resolution through an unambiguous viewing angle for cryo-EM could be to orient the carotenosomes on a functionalized lipid surface^56^, followed by acquisition of tilted image pairs. However, through the application of complementary restraints from LD, CD and ACD together with *ab initio* calculation, and by following our systematic procedure for searching conformational space for the symmetric structure, it was still possible to derive a reliable high-resolution structure of the CsmA baseplate.

In our derived structure for the CsmA baseplate the BChl *a* rings are almost coplanar and far apart with the shortest Mg–Mg distance of 15 Å (the second shortest is 23 Å) whereas for the LH2 and LH1 structure, the BChl *a* rings are stacking (Mg–Mg distance is ca. 8 and 9 Å, respectively)^7^,^8^. In the CsmA baseplate structure the BChl *a*'s with 15 Å intra-molecular distance form dimer units that are weakly coupled to each other as our anisotropic CD data suggest (Supplementary Methods for more details). This is further supported by the recently published hole-burning study showing that dimeric character dominates in spectroscopic properties of baseplates from *Cba. tepidum*^57^. Conversely, in the structure determined by solid-state NMR of BChl *c* aggregates, the porphyrine rings were shown to stack closely forming long rods^3^. This hints to a difference in the mechanism of energy transfer where energy is not transferred within the CsmA baseplate but along the BChl *c* rods, directly through the baseplate, which serves as a gate to transfer the energy to the FMO complex. We note that the H25 side chains for the dimer point away from each other but that light could possibly induce a conformational change in the geometry bringing the BChl *a* rings closer together by a rotation around bonds in H25 possibly facilitating more efficient energy transfer to FMO.

Many residues are conserved among different CsmA orthologous peptides in different photosynthetic species (Fig. 5c for visualisation and Supplementary Table 2). In particular, residues G24/H25/W26 near the binding site for BChl *a* are identical but also M21 and I17 one and two turns away, respectively, making hydrophobic interactions with BChl *a*. Several residues in the C-terminal end are either entirely conserved or strongly similar (N44/N46 and I43/R45/Y48, respectively), which could indicate interaction with the FMO protein as demonstrated previously^48^,^49^. Furthermore, we note that several residues in the hydrophilic groove in the CsmA baseplate (Fig. 4c) show strong similarity, in particular, negatively charged residues E19(D), E23(Q) and D34(E/Q) and positively charged residues R16(Q), and R45(Q) (where letter in the bracket denotes the alternative amino acid in other homologous species). As discussed above we observe putative salt-bridges between oppositely charged residues between residues from these two groups both within a rod and bridging two rods. Therefore, we argue that these interactions are important for the structural integrity of the baseplate. This is supported by the zero-length cross-linking results obtained for chlorosomes^58^ indicating that oppositely charged side chains on different monomers act as bridging interactions to form multimers suggesting a mechanism for the self-assembly of CsmA monomers in a baseplate structure. Conversely, the aliphatic residues in the less densely packed part of the hydrophobic groove and in the open part of the hydrophilic groove are not conserved among all species. We speculate that these more flexible regions could bind different carotenoid molecules. Finally, C-terminal residues, 49–59 have low degree of homology. This is understandable, since these residues are unstructured for the CsmA baseplate and hang as loose ends at the outer side of the baseplate. This exposure of the C-terminal in the structure is also supported by the finding that C-terminal residues are cleaved off proteolytically for this *Cba. tepidum* system and for most of the other homologous sequences discussed above^48^.

In this study, we have presented the first high-resolution 3D structure of the protein-pigment baseplate antenna from *Cba. tepidum* in its native *in situ* heterogeneous full organelle environment. This was accomplished through a structure characterization protocol, which might serve as inspiration to the study of other similar challenging systems, using atomic level structural information from solid-state NMR combined with global information from cryo-EM on the protein organisation and with information from isotropic and anisotropic CD spectroscopy on the orientation of the BChl *a* pigments. Our detailed structural study provides an important basis for further insight into an extremely efficient light-harvesting system. For example, our results reveal how the BChl *a* ligand plays an integral role in the self-assembly of CsmA and the atomic-resolution structure allows the mechanism of energy transfer in the antenna system to be studied in detail. The unusual structure also provides an inspiration for constructing artificial light-harvesting devices where the baseplate structure could acts as a template in an arrayed construction. Moreover, our structure can be used further for understanding CsmA–FMO complex interaction, which is fascinating regarding the recent idea of exploiting FMO in quantum computing^59^.

## Methods

### Sample preparation

*Growth of Chlorobaculum tepidum bchK mutant*. Cells of fully ^13^C,^15^N-labelled mutant bacteria were grown in closed 1 or 2 l bottles in modified medium using the procedures described by Frigaard *et al.*^15^. Uniform ^15^N labelling was obtained by substituting the ammonium acetate for an equimolar amount of sodium acetate in the original CL medium and by using ^15^NH_4_Cl as the sole nitrogen source. Uniform ^13^C labelling was performed using substituting the sodium bicarbonate and sodium acetate in the original CL medium for the fully ^13^C-labelled substrate equivalents (NaH^13^CO_3_ and Na^13^CH_3_^13^COO).

*Isolation of carotenosomes*. The procedure was performed as described by Frigaard *et al.*^15^. In brief, the cell pellet of a 2 l cell culture was resuspended in 50 ml of isolation buffer (50 mM Tris, 2 M NaSCN, 10 mM sodium ascorbate, 5 mM Na_2_EDTA, 0.5 mM phenylmethanesulfonyl fluoride, 1 mM 1,4-dithiothreitol, pH 8.0). In order to disrupt the cells the suspension was passed through a French press. Cell extract was clarified by centrifugation (13,000*g*, 20 min, 4 °C) and the supernatant was supplemented with 20% (w/v) sucrose. The cell extract with the sucrose was transferred to ultracentrifuge tubes and overlaid with isolation buffer containing 5% sucrose. The tubes were centrifuged at 270,000*g* for 2 h at 4 °C. The carotenosomes after centrifugation appeared as a dark-orange band floating on top of the solution. This band was removed and the carotenosome preparation was dialysed against water for at least 24 h prior to freeze-drying.

*Solid-State NMR spectroscopy*. Roughly 36 mg of freeze-dried, fully-labelled ^13^C,^15^N carotenosomes sample were packed into a 4 mm ZrO_2_ rotor. All NMR spectra were recorded on a BrukerAvance-II 700 MHz (16.4 T) NMR spectrometer equipped with a standard 4 mm triple-resonance magic-angle spinning (MAS) probe. All spectra were measured using 10.5 or 12 kHz spinning, 32.45 ms acquisition with 85 kHz SPINAL-64 (ref. 60) decoupling. Uniformly ^13^C,^15^N-labelled L-alanine was used as an external reference. Bruker Topspin was used for data processing and Sparky^61^ for data analysis.

Different combinations of 2D and 3D spectra were acquired for data analysis. In detail, 2D homonuclear ^13^C-^13^C DARR, heteronuclear NCA and NCO experiments with broad-banded version as well as 3D NCACX, NCOCX, CONCA, CANCO spectra were obtained. Details for those experiments have been described previously^20^. The 2D homonuclear ^13^C–^13^C DARR^62^ correlation spectrum (shown in Supplementary Fig. 4a) used for obtaining the long-range constraints, was recorded using a 200 ms mixing time. The spectrum was acquired at ca. 7 °C using 10.5 kHz MAS, a recycling delay of 3 s, 200 ppm spectral width in the indirect dimension with 400 increments and 92 scans per increment. The signal-to-noise ratio for the strongest peptide peaks were around 50.

### CD, ACD and LD

Isotropic CD spectra were acquired at both room temperature and 77 K using a Chirascan-plus spectrometer (Applied Photophysics Ltd, United Kingdom) with a 2-nm spectral bandwidth. The sample was diluted with glycerol (1:2 volume ratio) to obtain a good optical quality glass sample at low temperatures. The sample was placed in a temperature-controlled Optistat DN liquid nitrogen cryostat (Oxford Instruments, United Kingdom). The optical density in the 800-nm absorption peak was ∼0.3 at 77 K (5 mm optical path). CD spectra were back-calculated using *ab initio* and density functional molecular orbital methods^63^ applying exciton theory with calculated normal mode vibrations and calculated Franck-Condon (FC) factor (see Supplementary Methods for more details)^64^.

LD and ACD spectra were obtained from macroscopically aligned carotenosomes. To this end the samples were fixed in 5% polyacrylamide gel cubes (1 cm), diluted to optical density of 1 at 444 nm or 800 nm, for measurements in the visible and near-infrared region, respectively. The samples were oriented by compressing the gels uniaxially by a factor of 1.7. The particles are thus oriented preferentially with their smallest dimension parallel to the compression direction. Room temperature LD and ACD spectra were recorded using a Jasco 815 spectrometer. For LD measurements the gels were placed with the compression axis perpendicular to the beam propagation (‘edge' configuration), with linearly polarized light parallel or perpendicular to the compression axis. For ACD measurements the gels were placed with the compression axis parallel to the measuring beam (‘face' configuration). In this configuration LD is zero (see Supplementary Methods for more details).

*Freeze-fracture*. Fixed bacteria were equilibrated overnight in 25% glycerol at 4 °C, attached to the gold holders, and snap frozen in Freon 22 cooled in liquid nitrogen. *Cba. tepidum* bacteria were fractured in a Balzer's freeze-fracture apparatus (BAF 300; Balzers) at −100 °C. Samples were immediately rotary shadowed at an angle of 25 °C with 320 Hz thick layer of the platinum and carbon replicated 5 × 1 s. The replicas were cleaned overnight in 40% chrome oxide, rinsed with water, and analysed with a CM100 transmission electron microscopy (TEM) microscope (FEI) (Normal observations at 80 kV). Images were recorded by CCD camera 1 K MegaView III from (Olympus Soft Imaging Solutions), image handling by AnalySiS from the same company. A calibrated magnification of × 92,000 was used.

*Electron microscopy*. The grids used were 300 mesh Cu grids, coated with integral carbon and glow-discharged for 30 s. Samples for EM in negative stain and vitreous ice were prepared by adding 100 μl of 0.1 M cacodylate buffer and 50 μl of the *Cba. tepidum* spinning down the mixture with airfuge 273,000*g* in 10 min × 2. After removing the supernatant, the pellet was suspended with up to 200 μl cacodylate buffer. This procedure was repeated 3 times to get rid of sucrose.

Specimens for the negative staining EM were prepared by putting 5 μl of the specimen on EM grids and staining with 1% of uranyl acetate in aqueous solution for 1 min EM images were acquired with a FEI CM100 microscope operated at 80 kV. Data were collected at a magnification of × 64,000 with the CCD camera 1 K MegaWiev III from Olympus Soft Imaging Solutions. Image processing was carried out by AnalySiS from the same company.

Cryo-EM samples were prepared by vitrifaction in a FEI Vitrobot Mark II (TM). Sample of 10 μl was transferred to quantifoil R 2/4 grids, which have been glow-discharged for 30 s. Excess liquid was blotted off for 1 s and the sample was vitrified in liquid ethane slush. EM was performed at liquid nitrogen temperature on a FEI Titan-Krios Cryo-EM microscope operated at 300 kV acceleration voltage. Images were acquired under low-dose conditions (maximal dose: 5–8 e Å^−2^) at a magnification of 59,000 with a Gatan US4000 4k*4k CCD camera and FEI EPU software.

### Cryo-EM and calculation of 3D density model

The density model was calculated from a total of 1,750 individual extracted particles, yielding 48 class-averages (Supplementary Fig. 8) as performed with EMAN2 software. An initial model was obtained from a minimal set of three characteristic views using EMAN2 (ref. 37). A refined 3D model was calculated by aligning 1,750 individual particles using Strul^41^ to obtain a 3D reconstruction of these particles, using weighted back-projection in Spider^42^. Cryo-EM images were acquired at liquid N_2_ temperature under low-dose conditions (<5–8 e Å^−2^) at a magnification of 59,000 with a Gatan US4000 4k*4k CCD camera and FEI EPU software. All details can be found in the Supplementary Methods.

### Calculation of the baseplate structure

The structure of the CsmA baseplate was calculated applying a combination of solid-state NMR, Cryo-EM and isotropic and anisotropic CD through four phases ((i–iv)) using both existing software and new purpose-developed software (see below). An overview of the four phases is provided in Supplementary Table 3. (i) In the first phase, a set of candidate starting structures for the CsmA monomer with one BChl *a* coordinated to H25 was calculated using Xplor-NIH^65^ producing 368 structures, and the 64 with lowest energy were kept for the next phase. Standard simulated annealing procedures were used for this calculation, with a relatively low annealing temperature of 300 K to increase structural heterogeneity within the structure ensemble. The only source of experimental data used in this step was the constraints for the backbone dihedral angles inferred from the chemical shifts using TALOS+(ref. 43). Geometric force field parameters and partial charges for BChl *a* were taken from the Xplor-NIH parameters for Haem ligands in the similar skeleton part of BChl *a*, or taken from an *ab initio* calculation of BChl *a*^63^.

(ii) In the second phase, structure candidates for the full baseplate structure were calculated using our software, GASyCS (structure calculation using a Genetic Algorithm in Symmetry Constraints Space). The new software is described in Supplementary Methods (Supplementary Fig. 10). The monomer structure and the ambiguous distances constraints derived from a solid-state NMR DARR (200 ms mixing time, temperature set to 293 K) spectrum together with constraints derived using chemical shifts together with the shAIC potential^66^ was used to derive the structure in conjunction with constraints imposed by the symmetry of the baseplate. The spectra were acquired on a widebore Bruker Avance-II 700 MHz (16.4 T) spectrometer (see Supplementary Methods for more details). In addition, a distance constraint keeping the Mg-Mg distance between BChl *a* units below 15 Å was used along with a constraint ensuring the BChl *a* ring normal to be approximately orthogonal to the baseplate normal were used based on initial observations in isotropic CD spectra (see Supplementary Note 2). The 128 baseplate structure candidates having the lowest energy were further refined using Xplor-NIH and distance similarity constraints to enforce symmetric conformation using Xplor-NIH again. Here both the ambiguous distance constraints and the dihedral angle constraints derived using TALOS+ were used. This time a more advanced force field was applied using a classical Lennard-Jones potential for the Van der Waals interactions and a Coulombic force repulsive/attractive term for the electrostatics.

(iii) In the third phase, the 32 structural models from the above phase with the lowest hybrid constraint and force field energy were selected. The isotropic CD spectrum in the far-infrared region was back-calculated based on the structure. The structures having a similar shape as judged by a similar sign and a similar position for the sign inversion point were kept for the final refinement phase. The structures consistent with CD were overlaid with the cryo-EM density model using Chimera^67^.

(iv) In the fourth phase, the seven structural models from the previous phase compatible with CD and fitting the cryo-EM density best were selected. Xplor-NIH was used to refine the structures with a protocol similar to phase III, but this time using a simple repulsion term for the electrostatics. The cryo-EM density data was included in the final refinement applying the probDistPot potential^68^ with the cross-correlation option to optimize agreement with the cryo-EM derived density. Prior to the refinement, the structure was systematically translated and rotated to optimize initial overlap with cryo-EM density. After the refinement, one of the structural models produced structure ensembles with significantly lower energies compared with other ensembles (Supplementary Fig. 3a), and this model was regarded as having the correct fold. The structure calculation statistics are described in Supplementary Note 3. To increase convergence, and to iteratively further refine the structure, the same refinement was repeated using the best structure from the previous step. The CD spectrum was back calculated for the 10 (out of 80) structures with lowest energy and the structure with the best agreement with the CD spectrum was regarded as the *final* structure. The heavy atom coordinate r.m.s.d. for these 10 structures were 1.41 Å for residues 6–48 and BChl *a*.

Finally, the structure was validated; the anisotropic CD spectrum at room temperature and the isotropic CD spectrum at 77 K were also simulated using more detailed theory (as described in Supplementary Methods) and a good agreement between observed and predicted values was obtained (Fig. 3e, r.m.s.=0.0440 with normalized intensity), hence, validating the structure.

### Data availability

The atomic coordinates of final structure have been deposited in the Protein Data Bank under accession number 5LCB. The NMR chemical shifts have been deposited in the Biological Magnetic Resonance Data Bank, entry 34012, and the cryo-EM density model in the EMDB database under accession number, EMD-4033.

The software, GASyCS, for efficient geometry optimization of highly symmetric oligomeric structures, is available in Supplementary Information as Supplementary Data files. The data that support the findings of this study are available from the corresponding author on request.

## Additional information

**How to cite this article:** Nielsen, J. T. *et al.* *In situ* high-resolution structure of the baseplate antenna complex in *Chlorobaculum tepidum*. *Nat. Commun.* 7:12454 doi: 10.1038/ncomms12454 (2016).

## Supplementary Material

Supplementary Information Supplementary Figures 1-10, Supplementary Tables 1-4, Supplementary Notes 1-4, Supplementary Methods, Supplementary References

Supplementary Data 1 Python code for GASyCS main program

Supplementary Data 2 Python code for sub program used by GASyCS

Supplementary Data 3 Python utilities code used by GASycs

Supplementary Data 4 Python code used by GASyCS

Supplementary Data 5 README instruction file - all datasets 1-5 should be combined into one single zip file.

## Figures and Tables

**Table 1:** Restraint and structure statistics.

NMR distance & dihedral constraints	
*Distance constraints*	
Total distance constraints*	60
Intra-residue	3
Inter-residue	57
Sequential (|i−j|=1)	26
Medium range (|i−j|≤4)	22
Long range (|i−j|>4) or inter-chain	9
Dihedral angle	88
Average number of assignment possibilities per restraint	5.90
Average minimum effective distance† (Å)	5.79
	
*Structure statistics*	
Violations (number of/average)	
Distance constraints‡ (Å)	0/0.0
Dihedral angle constraints (°)	1/0.77
Deviation from idealized geometry	
Angle r.m.s. (°)	1.29
Bond r.m.s. (Å)	0.011
Ramachandran statistics§	
Most favourable regions	85.0%
Additionally allowed	11.7%
Generously allowed	2.2%
Disallowed regions	1.1%
Average pairwise r.m.s.d.|| (Å)	1.41

^*^Only the non-trivial constraints were used as defined in Supplementary Note 4.

^†^See Supplementary Note 4 for the definition of the effective distance.

^‡^As calculated by Xplor-NIH. Note that the distances calculated by Xplor-NIH differ slightly from the observed distances calculated as defined in Supplementary Note 4 (Supplementary Table 1), since Xplor-NIH sum over all intra/inter chain combinations including combinations related by symmetry.

^§^As calculated by PROCHECK^69^.

^||^Coordinate r.m.s.d. calculated for 10 ensemble members.
