# Supplementary material for: In situ high-resolution structure of the baseplate antenna complex in Chlorobaculum tepidum
Source: Nat Commun. 2016 Aug 18;7:12454. doi: 10.1038/ncomms12454 (PMC4992139; doi:10.1038/ncomms12454)
Supplement: Supplementary Information — Supplementary Figures 1-10, Supplementary Tables 1-4, Supplementary Notes 1-4, Supplementary Methods, Supplementary References [file ncomms12454-s1.pdf]

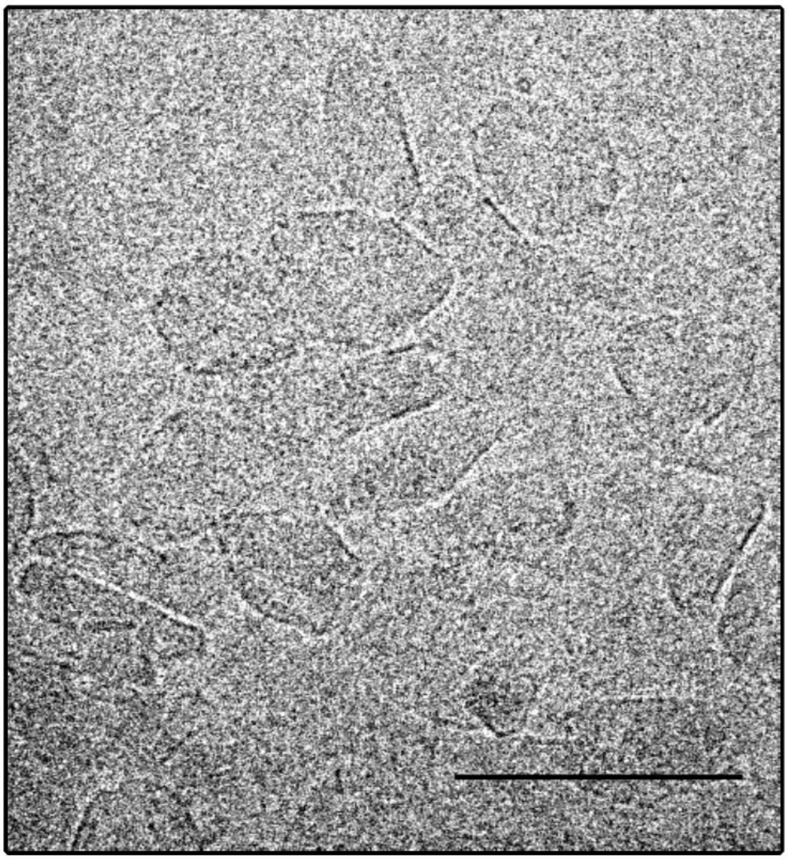

Supplementary Figure 1. TEM images of the carotenosomes Overview of unstained isolated carotenosomes of *Cba. tepidum* embedded in an amorphous ice layer prepared on quantifoil. The scale bars indicate 100 nm (see also Supplementary Note 1).

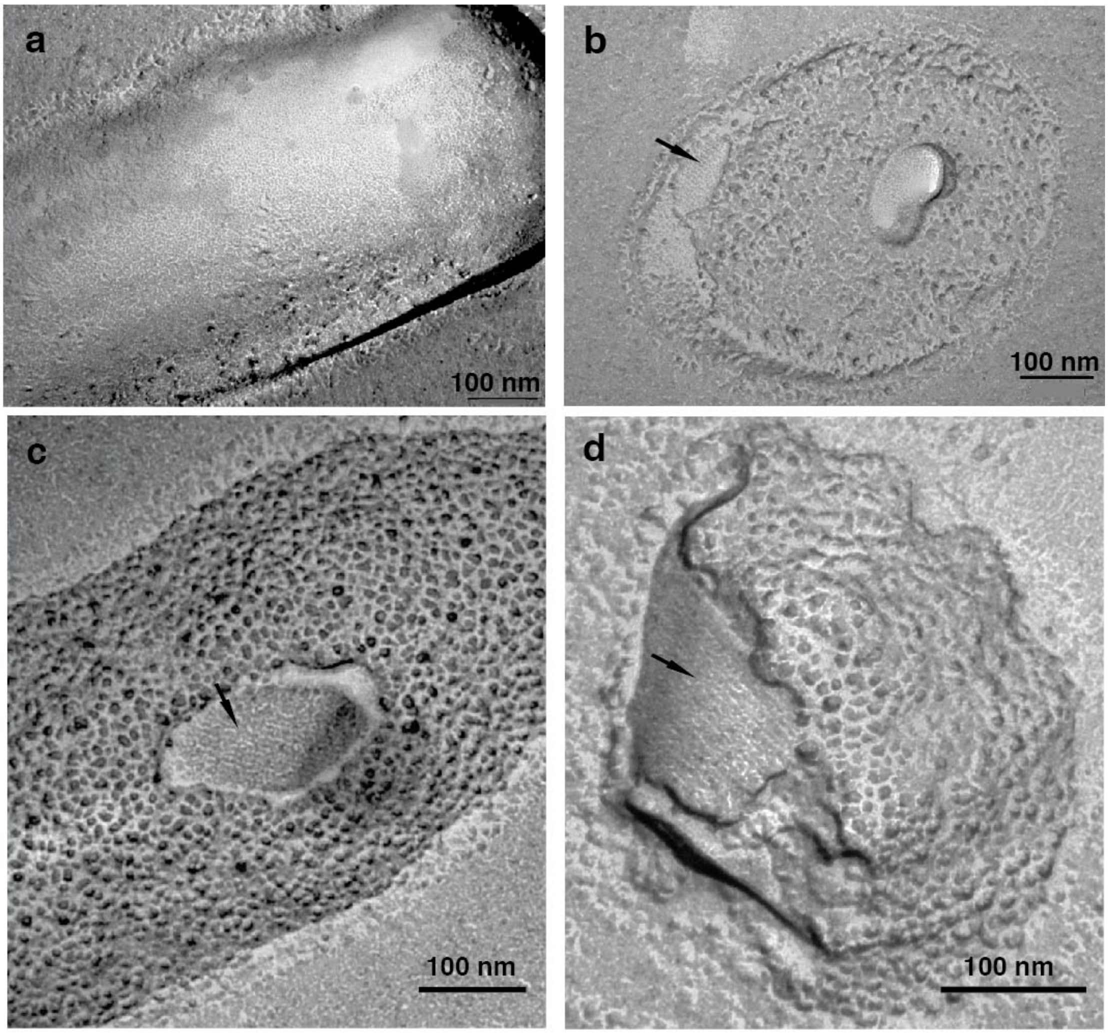

Supplementary Figure 2. **Freeze-fracture images of whole bacteria**, highlighting striations with black arrows. (**a**) The whole *Cba. tepidum* bacterium (wild type). (**b**) Example of the chlorosome with striation pattern. (**c**) Mutant *Cba. tepidum* showing carotenosomes with detectable striations originating from the baseplate, (**d**) enlarged region of *Cba. tepidum* mutant cell with distinguishable striation pattern due to crystalline substructure.

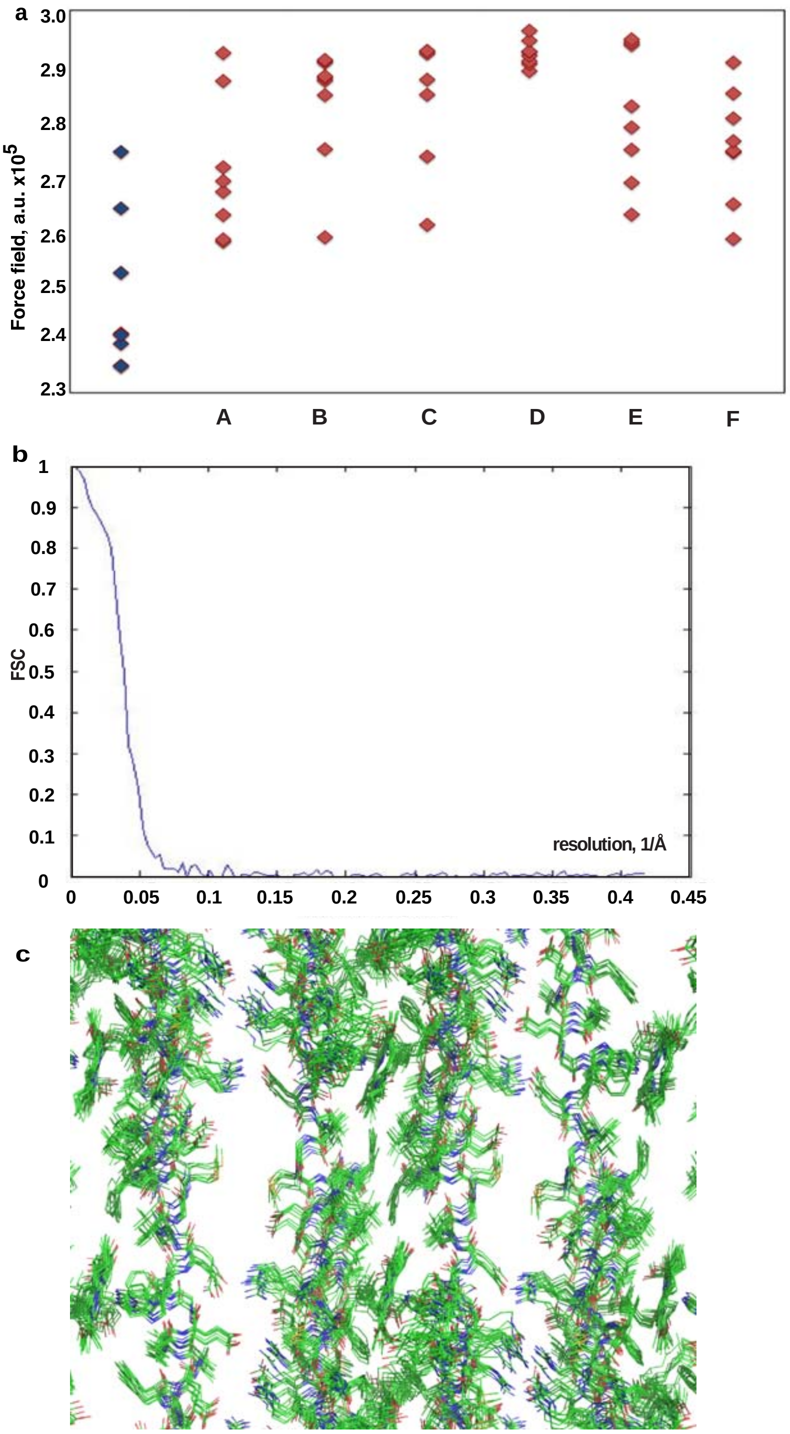

Supplementary Figure 3. **Structure Validation**. (**a**) Sum of Xplor-NIH force field and constraint energy showing the eight best structures out of 80 parallel calculations for each model. Our final structure, shown as blue diamonds, clearly represents the lowest post-refinement energy compared to six models with next best energies (red diamonds). The labelling on the x-axis corresponds to the numbering of items in Supplementary Figs 6 and 7. (**b**) Fourier shell correlation (FSC) curve of the Cryo-EM reconstruction model after a single round of refinement indicating a resolution of 25.5 Å (19.1 Å) according to the FSC=0.5 (FSC=0.143) criteria. (**c**) 10 lowest energy structures from structure refinement (see Supplementary Note 4).

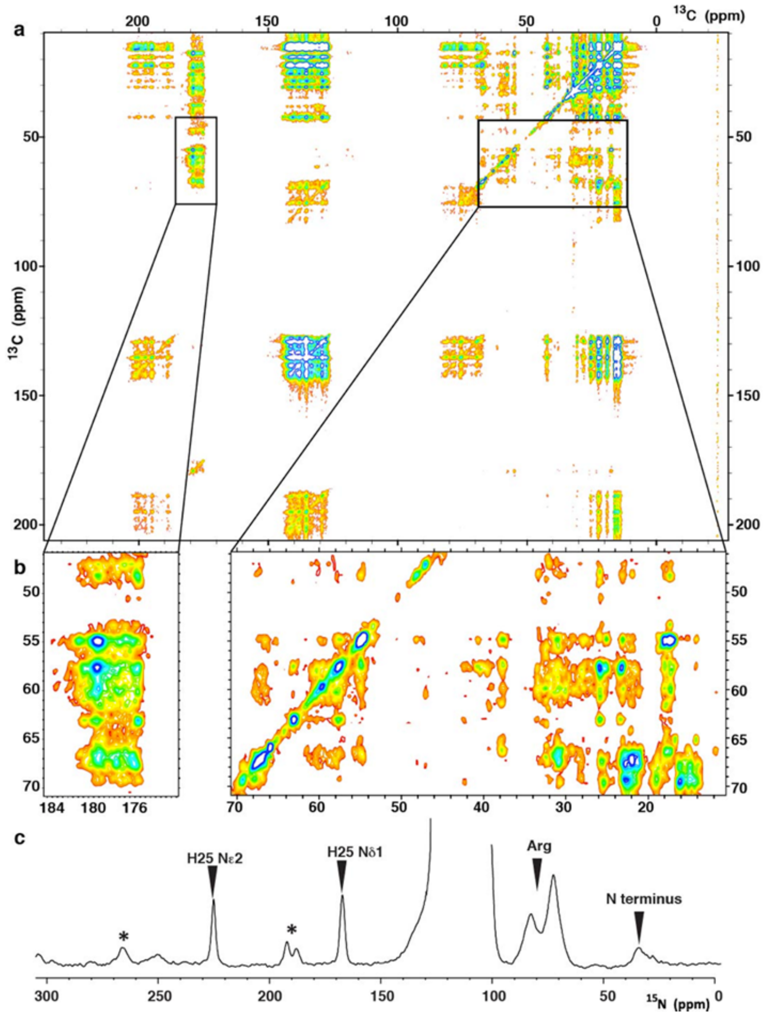

Supplementary Figure 4. Solid-state NMR spectra. (**a**) ^{13}C-^{13}C DARR spectrum (top panel) with the 200 ms mixing time containing both peaks from carotenoids (190-210, 120-150, and 5-50 ppm), lipids (5-50 ppm), and proteins (170-182, 45-70, and 5-50 ppm). (**b**) Protein regions are enlarged (bottom panel). (**c**) ^{15}N CP- MAS spectrum of the same sample with arrows indicating non-backbone resonances from the CsmA and stars represent BChl *a* signals.

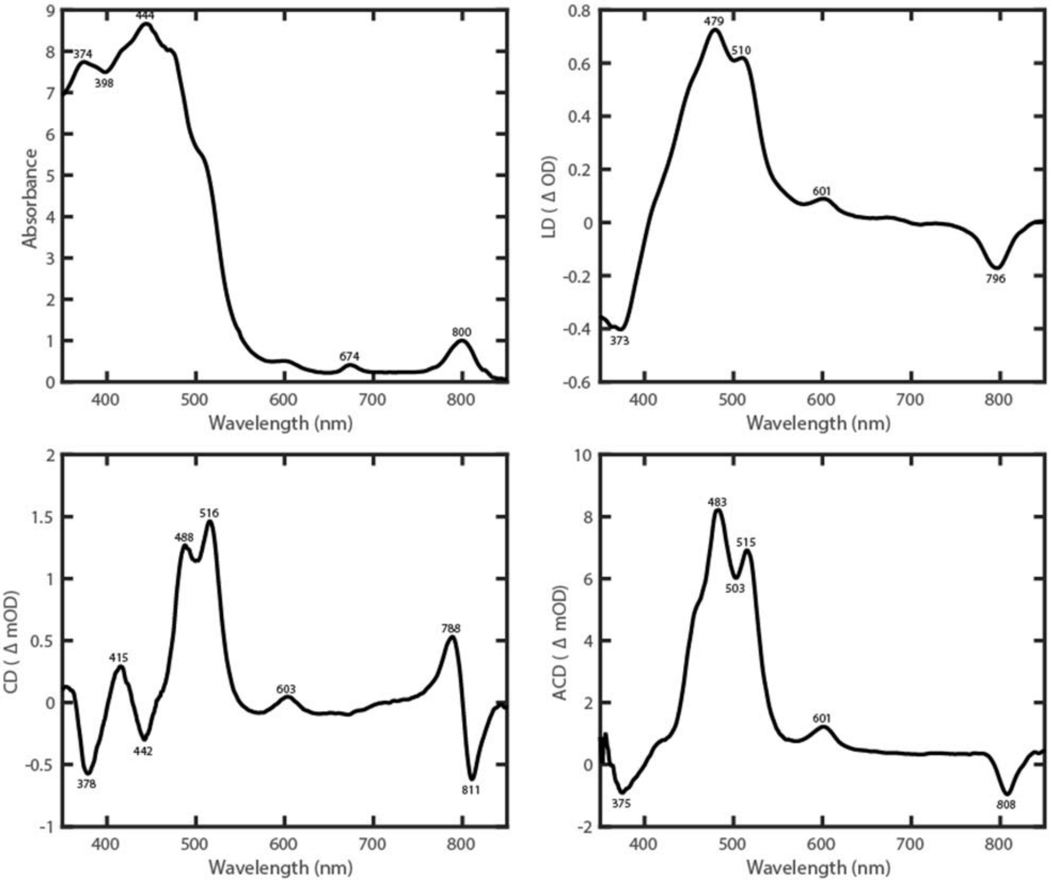

Supplementary Figure 5. Absorption, linear dichroism (LD), circular dichroism (CD) and anisotropic circular dichroism (ACD) spectra of carotenosomes, measured at room temperature. LD and ACD spectra were measured from macroscopically aligned carotenosomes with incident light parallel or perpendicular to the membrane plane, respectively. The spectra are normalized to absorbance of 1.0 at the Q_y maximum (see also Supplementary Fig. 9).

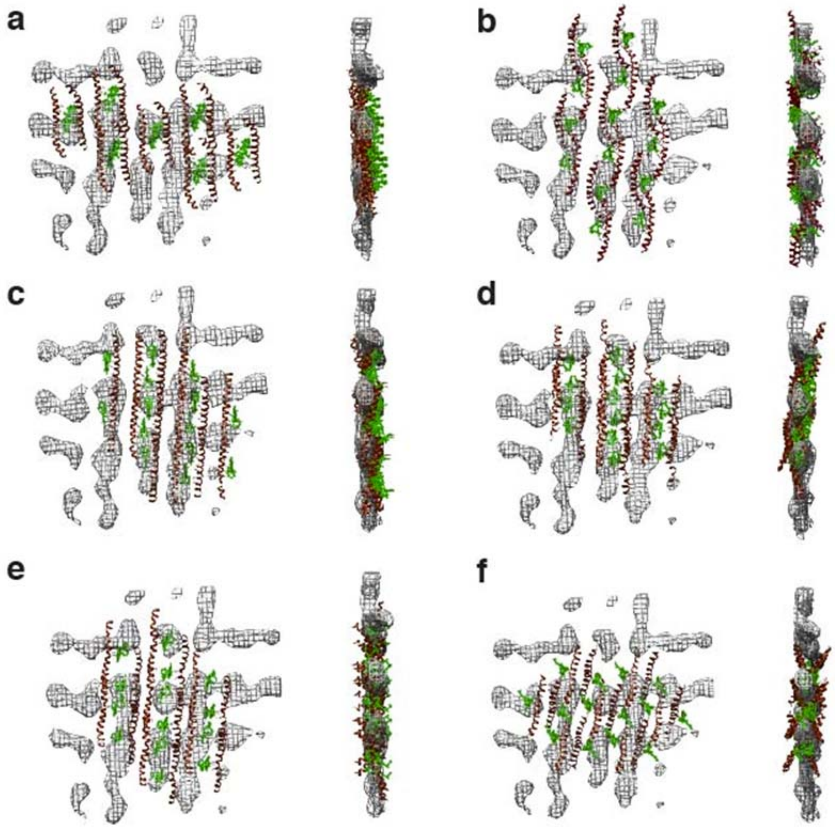

Supplementary Figure 6. Structure candidates before Cryo-EM refinement. Overlay of alternative structural models before refinement against the cryo-EM density showing the 6 best competing models for comparison with our final structure (main text, Fig. 3g,i,j). Only the structured regions residues 7-48 are shown for clarity. The labelling **a-f** corresponds to the labelling in Supplementary Figs 7 and 3a.

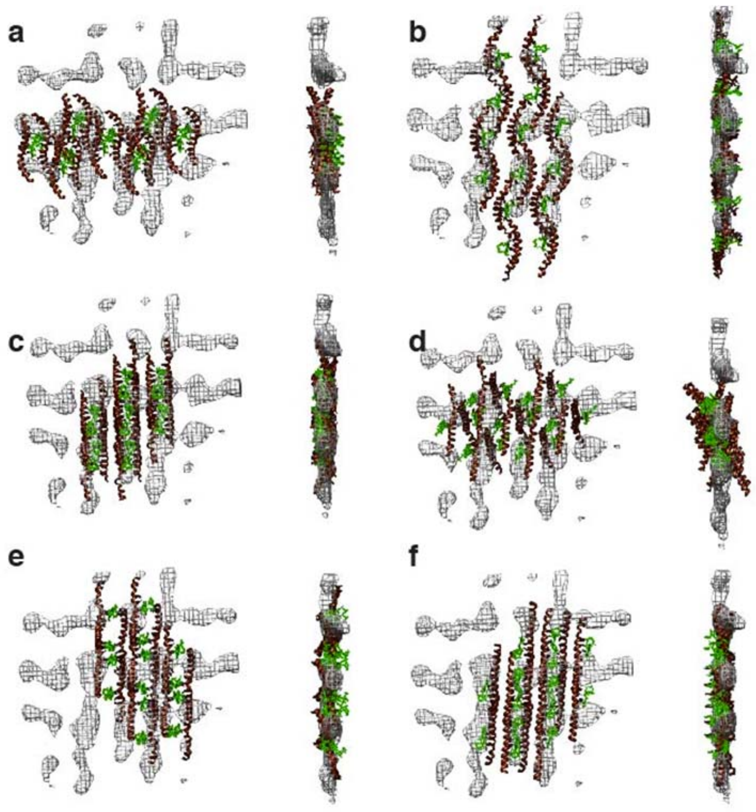

Supplementary Figure 7. Structure candidates after Cryo-EM refinement. Overlay of alternative structural models after refinement against the cryo-EM density. Showing is the six best competing models for comparison with our final structure (main text Fig. 3g,i,j). The labelling **a-f** corresponds to the labelling in Supplementary Figs 6 and 3a. The correlation function for agreement with cryo-EM density model is 0.149, 0.134, 0.153, 0.028, 0.130, 0.176 for models a-f, respectively, as calculated by Xplor-NIH[1]

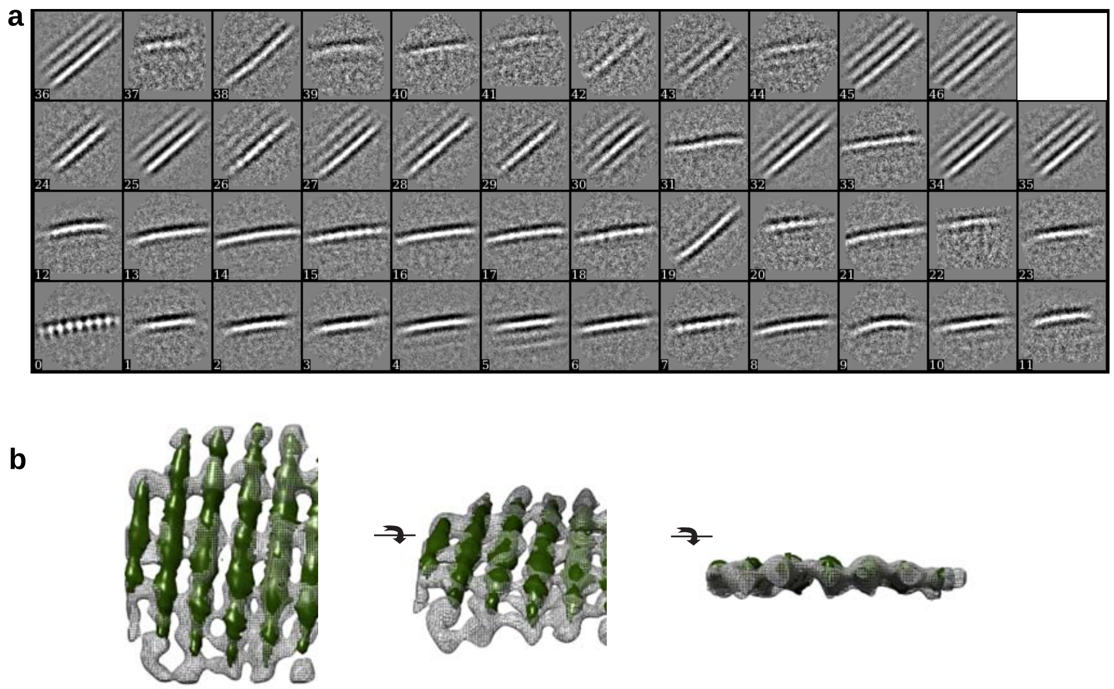

Supplementary Figure 8. Density model obtained by cryo-EM. (**a**) Class averages of ice-embedded selected baseplate particles. The 1750 individual particle images were classified and averaged in EMAN2. (**b**) Refined reconstruction model of the carotenosomes baseplate (gray mesh) overlapped with the initial model (green surface) shown at different projection angles.

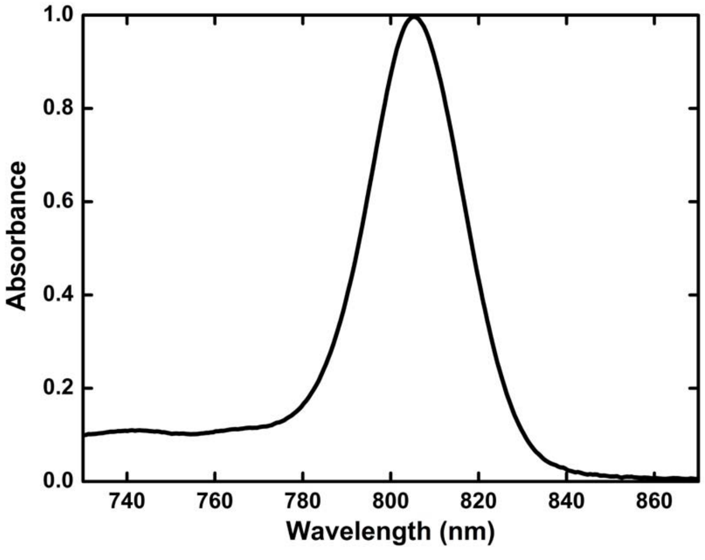

Supplementary Figure 9. Absorption spectrum of the carotenosomes from *Cba. tepidum* in the Q_y spectral region at 77 K. The spectra are normalized to absorbance of one at the Q_y maximum[2].

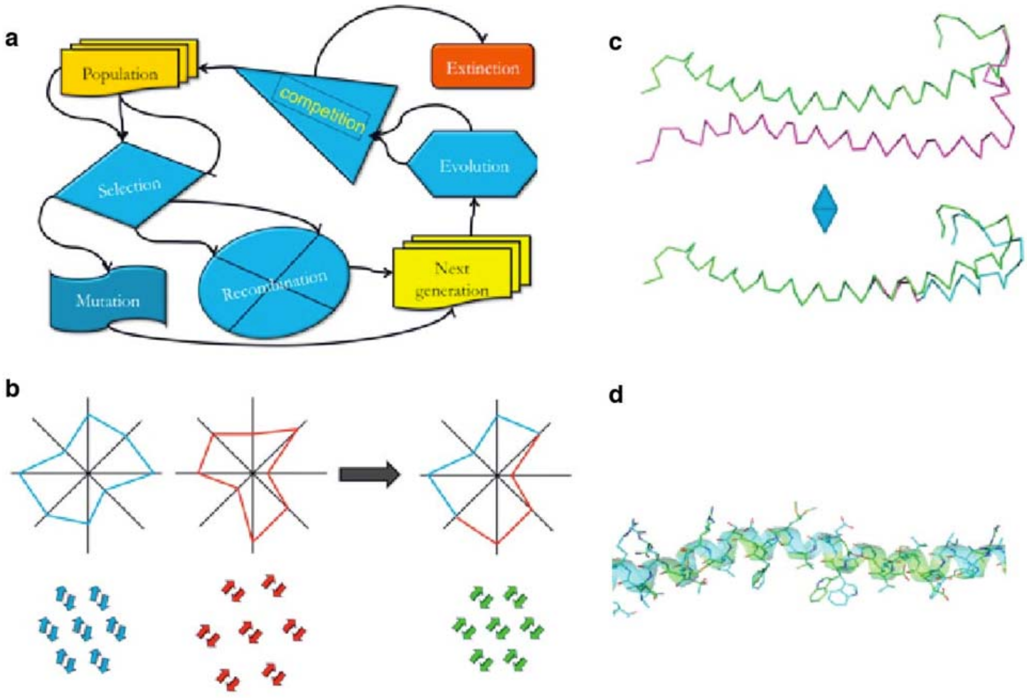

Supplementary Figure 10. Visualization of the GASyCS algorithm. (**a**) Overview of the events during a generation in the genetic algorithm. Illustration of genetic operations: (**b**) Symmetry recombination: The conformation of the two parents is shown to the left of the arrow in blue and red both as an 8-axis spider-diagram and as a cartoon. The result of recombination is shown right of the arrow in this example keeping genes encoding the dimer conformation from one (the red) parent and genes encoding the translation from the other parent. (**c**) Backbone double-crossover. Top view: the two parents shown as Cα-trace, bottom view: combined resulting operation (cyan) overlaid with the structures of the two parents (green and magenta, the latter only showed for the substitution window). (**d**) Overlay of a monomeric structure before and after a sequence of mutations, causing the side chain conformational changes (cartoon).

Supplementary Table 1: Summary of restraints and distances.

Δ_{min} [a]	Δ_{all} [b]	r_{eff}	assigment1 [c]	r_{obs1} [d]	assigment2	r_{obs2} ...	assigmentN	r_{obsN}
1	1	3.783	G24CA-H25CA	3.828	G24CA-W26CA	5.903		
1	1	4.173	L36CG-G37CA	4.605	K38CG-G37CA	4.774		
1	1	4.267	E23CB-G24CA	4.455	E27CB-G24CA	5.460		
1	1	4.597	F7CA-T8CB	4.752	F18CA-V22CA	6.117		
1	1	7.240	L11CD2-I10CA	7.268	K38CG-I10CA*	13.547		
2	2	4.889	T8CA-V6C	4.931	T8CA-A12C	8.047		
3	3	5.046	V29CG2-W26CA	5.082	V29CG2-F33CA	8.591		
5	5	10.307	V29CG2-G24CA	10.416	V6CG1-G24CA*	16.418		
IC	6	10.336	D34CB-T8CA*	10.933	D34CB-T40CB	12.733		
IC	17	9.331	L11CG-T28CA*	10.055	L11CG-T40CA*	11.057		
0	0	4.085	M21CE-CA	4.086	V29CG2-M21CA	13.268	V6CG1-M21CA*	17.657
1	1	3.730	M41CA-T40CA	3.836	W26CA-T28CA	5.623	K38CA-T40CA	5.807
1	1	3.743	D9CA-T8CA	3.802	L11CA-T8CA	5.794	L36CA-T40CB	7.417
1	1	4.184	D9CA-T8CB	4.382	L32CA-V29CA	5.409	L32CA-T28CA	7.571
1	1	4.606	M21CB-V20CA	4.887	E23CG-V20CA	5.911	M21CB-I17CA	7.096
1	1	6.927	M21CE-V20CA	7.258	M21CE-I17CA	8.919	M21CE-I43CA*	12.833
2	2	6.462	T28CG2-W26CA	7.100	T28CG2-H25CA	7.643	T28CG2-F33CA	10.168
4	4	6.297	M31CB-S35CA	6.331	M41CB-S35CA	11.841	E23CG-S35CA*	13.781
IC	4	7.260	W26CB-A47CA*	8.112	I43CG1-A47CA	8.244	E19CB-A47CA*	13.986
1	1	3.377	M21CA-V22CA	3.746	M21CA-V20CA	3.854	M21CA-I17CA	7.282

M21CA-I43CA* 10.254
1 1 3.580 N44CA-I43CA 3.741 A14CA-I17CA 5.310 A13CA-I17CA 5.406 N46CA-I43CA 5.823
1 1 4.462 F18CB-I17CA 4.909 E19CG-V20CA 5.714 E19CG-I17CA 6.419 F18CB-V20CA 6.594
1 1 4.726 T40CG2-G39CA 4.730 T8CG2-G24CA* 13.825 T8CG2-G39CA* 14.142 T40CG2-G24CA* 14.151
4 4 8.323 L11CD2-F7CA 8.795 K38CG-F18CA* 11.011 L11CD2-F18CA 12.736 K38CG-F7CA* 16.320
5 5 9.801 V29CG2-G24CA 10.416 V29CG2-G39CA* 12.456 V6CG1-G24CA* 16.418 V6CG1-G39CA* 18.362
IC 6 4.985 A12CB-S35CA* 4.991 M41CE-S35CA 12.829 I17CG2-S35CA* 13.616 V6CG2-S35CA* 15.169
IC 6 8.545 S35CA-T8CB* 9.381 S35CA-V29CA 10.536 S35CA-T28CB 12.264 S35CA-V22CA* 15.329
1 1 4.122 L11CB-A12CA 4.363 D9CB-A12CA 6.073 L11CB-A14CA 6.429 L11CB-A13CA 6.442 D9CB-A13CA 6.721
1 1 5.945 T8CG2-F7CA 6.115 L11CD2-F7CA 8.795 T40CG2-F18CA* 9.623 T8CG2-F18CA 15.504 T40CG2-F7CA* 16.639
1 1 6.326 L11CD2-I10CA 7.268 T8CG2-I10CA 7.351 L32CD2-I10CA* 9.178 L36CD2-I10CA* 10.665 T40CG2-I10CA* 13.935
2 2 6.942 I43CD1-M41CA 7.935 I43CD1-H25CA* 8.516 I43CD1-W26CA* 8.783 I43CD1-F33CA* 15.728 I43CD1-S50CB 15.830
4 3 3.954 I17CD1-A13CA 4.176 I17CD1-A14CA 4.890 I17CD1-N44CA* 17.453 I17CD1-N46CA* 17.486 I17CD1-M51CA* 24.858
4 3 3.954 I17CD1-A13CA 4.176 I17CD1-A14CA 4.890 I17CD1-N44CA* 17.453 I17CD1-N46CA* 17.486 I17CD1-M51CA* 24.858
4 4 4.838 M31CE-S35CA 4.860 A13CB-S35CA* 9.730 A14CB-S35CA* 10.480 A47CB-S35CA* 14.942 I43CG2-S35CA* 15.285
1 1 3.704 E27CA-T28CA 3.870 W26CA-T28CA 5.623 M31CA-V29CA 5.764 W26CA-V29CA 6.287 M31CA-T28CA 6.985 F33CA-V29CA 7.515
1 1 4.437 L11CG-A12CA 4.562 L11CG-A14CA 6.653 L11CG-A13CA 7.003 L11CG-N46CA* 14.696 L11CG-N44CA* 15.948 L11CG-M51CA* 21.317
1 1 6.055 I17CD1-R16CA 6.352 I17CD1-L11CA 8.463 I17CD1-D9CA 9.443 I17CD1-L36CA* 11.487 I17CD1-S35CB* 11.530 I17CD1-D34CA* 15.571
2 2 7.879 M41CE-G39CA 8.572 A12CB-G39CA* 9.571 A12CB-G24CA* 13.780 M41CE-G24CA* 14.011 V6CG2-G24CA* 16.440 V6CG2-G39CA* 18.019
4 4 4.837 M31CE-S35CA 4.860 A13CB-S35CA* 9.730 A14CB-S35CA* 10.480 I17CG2-S35CA* 13.616 A47CB-S35CA* 14.942 I43CG2-S35CA* 15.285
IC 10 8.614 D34CB-A12CA* 9.019 D34CB-A13CA* 12.519 D34CB-A14CA* 13.726 D34CB-N46CA* 14.517 D34CB-N44CA 16.539 D34CB-M51CA* 18.603
IC 6 9.504 E27CG-S35CA* 10.944 M41CG-S35CA 10.978 E27CG-V6CA* 14.911 M21CB-S35CA* 16.034 M21CB-V6CA* 17.042 M41CG-V6CA* 20.585
IC 9 5.062 A12CA-S35CA* 5.116 A13CA-S35CA* 8.564 A14CA-S35CA* 10.119 N46CA-S35CA* 14.730 N44CA-S35CA 15.692 M51CA-S35CA* 19.877
IC 13 8.887 V22CB-N46CA* 9.010 M31CG-N46CA* 15.290 M31CG-N44CA* 18.202 V22CB-M51CA* 18.848 I10CG1-N46CA* 18.951 M31CG-M51CA* 21.363
0 0 2.499 E23CG-CA 2.501 E23CG-E19CA 6.707 E23CG-W26CA 7.964 E23CG-M41CA* 9.959 E23CG-K38CA* 10.253 E23CG-M31CA 15.337 E23CG-S50CB* 18.196
1 1 6.005 T28CA-E27CD 7.017 V22CA-E23CD 7.289 T28CB-E27CD 7.819 V29CA-E27CD 9.493 T28CA-E23CD 12.863 T8CB-E27CD* 12.895 V29CA-E23CD 14.406
2 1 3.829 M31CA-V29C 4.508 S50CB-M51C 5.050 M41CA-N44CG 5.104 S50CB-Y48C 5.556 M41CA-I43C 5.710 F33CA-V29C 6.637 W26CA-V29C 7.282
IC 2 6.913 A14CB-G39CA* 8.369 M41CE-G39CA 8.572 I17CG2-G39CA* 9.564 A12CB-G39CA* 9.571 A47CB-G39CA* 10.848 I17CG2-G24CA 11.282 V6CG2-G39CA* 18.019
IC 7 8.433 A47CA-T40C* 9.297 A47CA-V22C* 10.889 A47CA-T28C* 13.238 A47CA-G30C* 13.747 A47CA-G15C* 13.814 A47CA-S35C* 14.374 A47CA-I10C* 16.969
4 4 7.816 I43CG1-A47CA 8.244 V29CB-A47CA* 10.291 K38CB-A47CA* 13.396 M21CG-A47CA* 15.656 V20CB-A47CA* 16.283 I17CG1-A47CA* 17.597 I10CG1-A47CA* 19.151 V6CB-A47CA* 21.576
0 0 3.846 L11CD2-CA 3.862 V29CG2-L32CA 7.329 V6CG1-L11CA 11.572 V6CG1-L32CA* 13.141 V29CG2-R16CA* 13.799 V6CG1-S35CB* 14.038 V6CG1-R16CA 15.747 V29CG2-S50CA* 16.355 V6CG1-S50CA* 26.702
1 1 3.631 G15CA-R16CA 3.784 G52CA-S50CA 5.553 G30CA-L32CA 5.646 G15CA-L11CA 6.124 G30CA-D34CA 7.003 G30CA-S35CB 8.827 G30CA-L36CA 11.136 G30CA-D9CA* 11.194 G30CA-L11CA* 11.787
1 1 4.408 K38CB-G39CA 4.533 V22CB-G24CA 6.922 V20CB-G24CA 7.362 M21CG-G24CA 7.958 V29CB-G24CA 10.909 K38CB-G24CA* 11.198 I17CG1-G24CA 12.121 M31CG-G24CA 13.291 I10CG1-G24CA* 14.809 V6CB-G24CA* 16.276
4 3 5.809 M41CG-G37CA 6.047 E27CG-G30CA 8.292 M41CG-G15CA* 9.971 M21CB-G15CA 10.022 E27CG-G15CA* 12.870 M41CG-G30CA* 15.274 M21CB-G37CA* 15.283 M21CB-G30CA 16.694 M41CG-G52CA* 16.861 E27CG-G52CA* 21.778 M21CB-G52CA* 24.589
4 4 5.572 A14CA-F18CA 5.864 A13CA-F18CA 7.906 A12CA-F7CA 9.295 A13CA-F7CA 9.837 A12CA-F18CA 10.129 A14CA-F7CA 10.844 N44CA-F18CA* 12.353 N46CA-F18CA* 13.224 N46CA-F7CA* 18.994 N44CA-F7CA* 20.719 M51CA-F18CA* 21.099 M51CA-F7CA* 25.641
IC 5 6.544 G39CA-A14CA* 7.674 G39CA-A12CA* 8.442 G39CA-A13CA* 8.744 G39CA-N44CA 9.650 G24CA-N46CA* 11.176 G39CA-N46CA 11.942 G24CA-A14CA* 12.647 G24CA-A13CA* 13.327 G24CA-N44CA* 13.673 G24CA-A12CA* 14.158 G39CA-M51CA 18.118 G24CA-M51CA* 21.261
1 1 5.414 L11CD2-A12CA 5.712 L11CD2-A14CA 8.140 L11CD2-A13CA 8.437 K38CG-A12CA* 8.709 K38CG-A13CA* 10.285 K38CG-N46CA* 11.161 K38CG-A14CA* 11.240 K38CG-N44CA 12.345 L11CD2-N46CA* 14.645 L32CD1-N46CA* 15.496 L11CD2-N44CA* 16.230 K38CG-M51CA* 17.438 L11CD2-M51CA* 20.996 L32CD1-M51CA* 21.276
1 1 5.440 L11CD2-A12CA 5.712 T8CG2-A12CA 7.309 V29CG2-N46CA* 9.999 V29CG2-A14CA* 10.929 V29CG2-A12CA* 11.421 V6CG1-A13CA 11.826 V6CG1-A12CA 12.237 V29CG2-A13CA* 12.875 V6CG1-A14CA 13.621 V29CG2-N44CA* 14.031 V29CG2-M51CA* 18.970 V6CG1-N46CA* 21.550 V6CG1-

			N44CA* 23.002 V6CG1-M51CA* 28.414
IC	2	5.327	L36CD2-G15CA* 6.356 T40CG2-G15CA* 6.702 L32CD2-G30CA 7.597 T8CG2-G30CA* 7.600 L11CD2-G15CA 7.747 L11CD2-G30CA* 9.893 L36CD2-G30CA* 11.168 L32CD2-G15CA* 11.654 T8CG2-G15CA 11.826 T40CG2-G30CA* 14.146 T40CG2-G52CA* 18.206 L36CD2-G52CA* 19.650 L32CD2-G52CA* 21.930 L11CD2-G52CA* 22.121 T8CG2-G52CA* 23.911
4	4	5.259	F18CB-A14CA 5.539 F18CB-A13CA 8.164 L32CB-A12CA* 8.636 E19CG-A14CA 9.545 E19CG-A13CA 9.836 F18CB-A12CA 10.026 E19CG-N44CA* 10.401 E19CG-A12CA 10.643 E19CG-N46CA* 11.588 L32CB-A13CA* 11.646 F18CB-N44CA* 11.668 L32CB-A14CA* 12.124 F18CB-N46CA* 12.712 L32CB-N46CA* 14.686 L32CB-N44CA* 17.414 E19CG-M51CA* 18.897 F18CB-M51CA* 20.754 L32CB-M51CA* 20.763
4	4	6.322	T8CG2-A12CA 7.309 T40CG2-N44CA 8.456 T40CG2-A14CA* 9.920 L36CD2-A13CA* 9.924 T8CG2-A13CA 9.939 T40CG2-N46CA* 10.288 T40CG2-A12CA* 10.644 L36CD2-N46CA* 11.649 L32CD2-A13CA* 11.690 T40CG2-A13CA* 11.794 L36CD2-N44CA 12.710 L32CD2-N46CA* 14.809 T40CG2-M51CA* 16.250 T8CG2-N46CA* 16.259 L32CD2-N44CA* 17.333 L36CD2-M51CA* 18.297 T8CG2-N44CA* 18.650 L32CD2-M51CA* 20.988 T8CG2-M51CA* 22.808

[a] residue number difference, among the assignment possibilities, leading to the smallest effective distance, "IC" denotes an inter-chain assignment

[b] minimum residue number difference among the assignment possibilities

[c] effective distance, r_{eff} , as defined in the equation above

[d] assignment where "*" denotes an inter-chain assignment reported where the corresponding distance was shorted than the corresponding intra-chain distance. The set of assignment candidates was generated by in-house developed software used previously.[3]

Supplementary Table 2. Alignment of homologous sequences. Alignment using Clustal 2.1[4]. Conserved, homologous and weekly homologous residues are indicated by “*”, “:” and “.”, respectively. The names of the species are shown in the top in the same order as the corresponding sequences. The *Cba. tepidum* species studied here is shown in the first line highlighting conserved residues with bold letters.

```
CsmA NP 662819 Chlorobium tepidum TLS
CsmA_YP_912715_Chlorobium_phaeobacteroides_DSM_266
CsmA_YP_001960581_Chlorobium_phaeobacteroides_BS1
CsmA_YP_001944148_Chlorobium_limicola_DSM_245
CsmA_YP_001129854_Chlorobium_phaeovibrioides_DSM_265
CsmA_YP_002016619_Prosthecochloris_aestuarii_DSM_271
CsmA_YP_374196_Chlorobium_luteolum_DSM_273
CsmA_YP_002019351_Pelodictyon_phaeoclathratiforme_BU-1
CsmA_ZP_01386130_Chlorobium_ferrooxidans_DSM_13031
CsmA_YP_001995207_Chloroherpeton_thalassium_ATCC_35110
CsmA_YP_002462567_Chloroflexus_aggregans_DSM_9485
CsmA_YP_001633769_Chloroflexus_aurantiacus_J-10-fl
CsmA_OSCT_1087mod_Oscillochloris_trichoides_DG-6

--MSGGGVFTDILAAAGRIFEVMVEGHWETVGMLFDSLGKGTMRINRNAYGSM---GGG-SLRGSSPEVSGYAVPTKEVESKFAK
--MSGGGVFTDILAASGRIFEVMVEGHWATVGMLFDSLGKGVMRINQNAYGNMG--GGGGSLRGSSPEVSGYAVPSKAVESKFAK
--MSGGGVFTDVLAASGRIFEVMVEGHWATVGMLFDSLGKGVARINQNAYGVGG--GGGGSLRGSSPEVSGFAVPTKAVESKFAK
--MSGGGVFTDILAAAGRIFEVMVEGHWETVGMLFDSLGKGTMRINRNAYGNM---GGGGSLRGSSPEVSGYAVPTKAVESKFAK
--MSGGGVFTDILAAAGRIFEVMVEGHWETVGMLFDSLGKGTMRINRNAYGNM---GGGASLRGGSPEVSGYAVPTKAVESKFAK
--MSGGGVFTDILAAAGRIFEVMVEGHWETVGMLFDSLGKGTMRINRNAYGNLGG-GGG-SLRGSSPEVSGFAVPTKAVESKFAK
--MSGGGVFTDILAAAGRIFEVMVEGHWETVGMLFDSLGKGTMRINRNAYGSM---GGGTSLRGSSPEVSGYAVPSKAVESKFAK
--MSGGGVFTDILAAAGRIFEVMVEGHWETVGMLFDSLGKGTMRINRNAYGST---GGGTSLRGSSPEVSGYAVPSKAVESKFAE
--MSGGGVFTDILAAAGRIFEVMVEGHWETVGMLFDSLGKGTMRINRNAYGSM---GGGTSLRGGSPEVTGYAVPSKEVASKFAK
MPVGGGGAITEVLSSIGRISELMFEGHWYAFSKAVESLAQGSLRLNQNLWGSMGGGVGGSSLRGSSPSRE-YG---KQIESKFAK
--MATRGWFSESSAQVAQIGDIMFQGHWQWVSNALQATAAAVDNINRNAYPGVSRSGSGESSFGSS----GNVFRPKRIRSRFNR
--MATRGWFSESSAQVAQIGDIMFQGHWQWVSNALQATAAAVDNINRNAYPGVSRSGSGEGAFSSSP---SNGFRPKRIRSRFNR
--MATRGWFSESAGQVAQIGDIMFQGHWQWVSNALQATSAAVETINRNAYPGISRSGSGEGSFSAPTVSS---FRPKRIRSRFNR
  :.  * :::  .  .:* ::*.:***  ..  .:: . .   :*:* :       .* .  ...          * : *:* .
```

Supplementary Table 3: Summary of full structure derivation process

	Restraints types	Software employed	Number of structures / kept structures[a]

**Phase I**	Dihedral angle	Xplor-NIH[5,6], Talos+[7]	368 / 64
**Phase II**	Distances, chemical shifts	GASyCS, shAIC[8], Xplor-NIH	128 / 32
**Phase III**	Agreement with CD spectrum and density model	*ab initio* and density functional molecular orbital methods[10,20], Chimera[9]	n.a.[b] / 7
**Phase IV**	Distances, dihedral angle, density model	Xplor-NIH with probDistPot[1]	7*80/1[c]

[a] Number of structures calculated in the phase / number of structures kept as starting point for the next phase

[b] No new structures were calculated in this phase. The 32 structures from the previous phase was used a starting point.

[c] 7 parallel calculations, each run generating 80 structures, starting from the 7 best structures from the previous phase.

Supplementary Table 4: GASyCS generation control parameters

VDW definition[a]	Mode[b]	Genetic operations[c]	Output size[d]	f_{Lim}[e]	Evolution optimization[f]	Final size[g]	w_{Div}[h]
CG[i]	initialization[j]	randomsymm.[k]	256	-	SA[l]	128	5.0
CG	append[m]	SR[n]	384	25	SA	256	5.0
CG	append	SR	768	30	SA	512	5.0
CG	initialization	-	-	-	Min[o]	394	5.0
CG	append	SR + BR[p]	-	20	SA	256	5.0
CG	append	SR + BR	.	25	Min	256	5.0
CG	append	SR + BR	.	25	Min	256	4.6
CG	append	SR + BR	.	25	Min	256	4.2
CG	append	SR + BR	.	25	Min	256	3.8
HA[q]	initialization	-	-	-	SA + Min	192	4.0
HA	replace[r]	SR + BR + Mut[s]	-	16	Min	112	4.0
HA	replace	SR + BR + Mut	-	16	Min	64	3.5
HA	replace	SR + BR + Mut	-	16	Min	32	3.0
HA	replace	SR + BR + Mut	-	16	Min	16	2.5
FA[t]	initialization	-	-	-	SA + Min	16	-
FA	replace	SR + BR + Mut	-	16	Min	12	3.5
FA	replace	SR + BR + Mut	-	16	Min	10	3.0
FA	replace	SR + BR + Mut	-	16	Min	9	2.5
FA	replace	SR + BR + Mut	-	16	Min	8	2.0

[a]Atomic representation used as basis for the Van der Waals energy term, E_{VDW}

[b]Procedure for including the new offspring in the population

[c]Genetic operations for generating new offspring

[d] Maximum size, s_{MAX}, of new generation

[e] Offspring generation is terminated when either the size of the population, *P*, exceeds s_{MAX} or the number of genetic operation executions for this operation exceeds $P*f_{Lim}$

[f] Procedure for optimizing the structure (evolution of the individual)

[g] Final size of the population after extinction
[h] Weight for diversity (see Supplementary Methods)
[i] Coarse grain representation. An average position of all heavy atoms in a residue is used in the definition of VDW. The distance is calculated between averaged positions and for the threshold the sum of the generalized VDW radius is used, which is defined as the standard deviation of all atom positions in the residues from the average position.
[j] No new offspring, but the fitness (the energy) is redefined through initialization either from library of pre-calculated monomer structures or through new VDW definition.
[k] Random values are drawn for the genes encoding the symmetry. The backbone and side-chains are fixed starting from the pre-calculated structures.
[l] Simulated Annealing
[m] The new offspring is added to the existing population
[n] Symmetry recombination (see Supplementary Methods, available in the online version)
[o] Simplex minimization
[p] Backbone recombination (see Supplementary Methods)
[q] The heavy atoms only are used.
[r] The new offspring replaces the parent with highest energy immediately
[s] Mutation of the side-chain geometry
[t] Full atom representation. Chemical shift pseudo energy calculated by shAIC is used in this phase (see Methods section).

Supplementary Note 1: Electron microscopy

The overall shape and size of isolated carotenosomes can be studied with transmission electron microscopy either by negative staining or by ice embedded samples. This study takes advantages of both EM techniques with the aim of exposing structural features of the long-range organization of the CsmA proteins. Negative staining, however, has one drawback, because it enhances the contrast of the water-accessible surface – small negative-stain clusters do not penetrate to the hydrophobic interior[10] Conversely, cryo-EM of ice-embedded samples provides a full set of cross-sections of the molecular complex structure. However, negative staining is a relatively simple way to check sample preparation and this method was therefore used here prior to cryo-EM.

Initial negative-staining studies revealed high tendency of carotenosomes to aggregate, until the sample preparation protocol was optimized (see Sample preparation). Examples of negative-stained carotenosomes of *Cba. tepidum* are shown in Fig. 1f (main text). These pictures show overall shapes and sizes as well as provide a good measure of sample preparation quality. The images reveal that the carotenosomes have approximately elliptical shapes with a relatively smooth surface. Even though the sample preparation protocol was optimized for the TEM study, carotenosomes exhibit a tendency of sticking to each other, as seen in Fig. 1f.

Cryo-EM of ice-embedded samples of isolated carotenosomes of *Cba. tepidum* reveal more detailed molecular complex structure. An overview of carotenosomes with different size and shape variations are shown in Supplementary Fig. 1. A collection of those images was used for further data analysis.

In order to dispel doubts about similarities of chlorosome and carotenosomes baseplate, freeze-fracture electron microscopy was used. For this purpose, the wild type and mutant *Cba. tepidum* bacteria were carefully examined. Supplementary Fig. 2 depicts galleries of images for wild type and mutant bacteria with enlarged chlorosomes and carotenosomes. Some of the fractures show supramolecular organization of protein rows, originating from the baseplate. The ridges of the baseplate lattice are perpendicular to the longitudinal axis of the chlorosomes and carotenosomes.

Further analysis of the power spectra of striated areas was also done for both carotenosome and chlorosome baseplates. The statistical analysis exhibits two identical repeating patterns in two perpendicular directions: 6 nm along the long axis and 3.3 nm along the transversal axis for both, chlorosomes and carotenosomes. A similar result was observed for the chlorosome in Chlorobium limicola[11]. As the Chlorobi species contains the FMO protein located between the baseplate and the inner membrane, the 6 nm spacing is proposed to originate in FMO complexes as discussed elsewhere[12,13]. Thus, comparison of the wild type *Cba. tepidum* chlorosomes with the mutant carotenosomes reveals basically the same baseplate (Supplementary Fig. 2).

Supplementary Note 2: Baseplate organization and symmetry

From previous solid-state NMR investigations, we could, based on secondary chemical shift data, conclude that the CsmA monomers *in situ* in the baseplate adopt a largely α-helical structure encapsulated into a fully symmetric baseplate super-structure[14]. Furthermore, we note now that a significant downfield shift to 225 ppm (see Supplementary Fig. 4c) for the $^{15}N\varepsilon_2$ chemical shift is indicative of Mg^{2+} coordination[15] and similar side chain chemical shifts were found for the Mg^{2+} coordinating Histidines in LH2[16,17]. Moreover, a significant deviation in H25 side chain $C\delta_2$ and $C\varepsilon_1$ ^{13}C chemical shifts from average values is indicative of protonation of Nδ1 and deprotonation of $N\varepsilon_2$[18] and coordination of Mg^{2+}, hereby demonstrating a

coordination of the BChl *a* ligand to H25. This coordination is a 1:1 coordination since we only observe one set of chemical shifts for H25 and the Bchl *a* resonances.

CD spectra were recorded, as described in the methods section, of a couple of different carotenosome samples, both at room temperatures (RT, 295 K) (Fig. 3f, main text) and at 77 K (Fig. 3e, main text). The spectra all have identical shape of CD bands in the baseplate absorption region around 800 nm, but a variation in CD signal intensities was observed. In the baseplate absorption region the shape of the CD spectrum was generally S-shaped, which is due to that two nearby CD bands of opposite sign overlap. This is indicative of weak excitonic interactions between pigments of the baseplate BChl *a* -complex. In the different samples, the maximum value of the so-called g-factor, which is the ratio of the CD intensity to the total absorbance of the sample[19], vary from about 0.0001 to 0.0008 at RT (0.0002 to 0.0012 at 77K). This indicates a structural heterogeneity between the samples. Based on our present CD spectrum calculations the magnitude of the g-factor seems to depend strongly on the mutual orientations of bacteriochlorin skeletons in the baseplate BChl *a* complex. This result suggests that there might be small orientation differences of bacteriochlorin skeletons between different samples

It has been shown, based on three dimensional linear dichroism analysis, that the BChl *a* molecules needs to be oriented with the bacteriochlorin plane approximately orthogonal to the plane of the baseplate[20] since the total transition dipole vector of the baseplate BChl *a* oligomer is oriented at ca. 77 degrees relative to the total transition dipole vector of chlorosome antenna (BChl *c* oligomers), which is stacked along the baseplate normal. Our LD results for carotenosomes support this architecture of pigments (See Supplementary Note 3). We also note that trial structures, which were calculated that violated this assumption and had BChl *a* rings with the ring plane parallel to the baseplate plane were not consistent with the observed CD spectra. A trimer or higher symmetry translated along the rotation axis (as in a triple-helix) would violate this observation. Furthermore, assuming that the repeating unit is indeed a dimer, if this dimer were translated along the rotation axis the two sides of the baseplate would be identical, which does not fit with earlier observations. If the dimer was translated at a general direction it would break the full symmetry, and hence only the proposed symmetry is possible. The following observations further support our proposed symmetry, both RT (Fig. 3f, main text) and 77 K CD (Fig. 3e main text) spectra shows only two CD bands in the baseplate absorption region due to

transitions from two different electronic states. There are not any fine structures observed at low temperatures. Also, 77 K and RT absorption spectra show only one absorption band without shoulders in region of 700 - 900 nm. Extra bands would mean closer contacts in higher order aggregates, i.e. smaller distances between more than two BChl *a* ring skeletons. These observations altogether suggest that the repeating unit is a dimer and that there are only weak inter-dimer interactions, because additional bands are not observed at low temperatures.

Occurring of this weak inter-dimer interactions are according in our ACD calculations; ACD calculations for a single BChl *a* dimer geometry from our structure calculation of the baseplate gave two ACD bands, disagreeing with the experimental observation of a single band. But for larger oligomeric BChl *a* baseplate structures ACD calculation gave only one negative ACD band as in the experiments. Because in the baseplate model structure an intra-dimer distance is much shorter than inter-dimer distances, intra-dimer exciton interactions are more stronger than inter-dimer interactions, and thus the dimeric character dominates in spectroscopic properties of the baseplate as experimental hole-burning study has also shown[21]

Supplementary Note 3: Analysis of LD and ACD spectra

LD and ACD spectra of oriented carotenosomes are plotted in Supplementary Fig. 5 in comparison with absorption and CD spectra of isotropic solution. The negative LD band in the BChl Q_y region signifies that the main absorbing transition dipoles are predominantly perpendicular to the membrane plane, whereas the Q_x transition (600 nm) is polarized parallel to the plane. The strong positive LD signal in the blue region (479 nm, 510 nm peaks) is evidence that carotenoid molecules are not randomly oriented but are parallel to the membrane plane. The degree of orientation relative to the BChl Q_y dipoles can be estimated by comparing the ratio LD/Abs in the blue and near-infrared region. The LD/A values at 440 nm and at 800 nm are both 6% of the theoretical values for perfect in-plane and out-of-plane order, respectively, the carotenoid S_2 and BChl Q_y transition dipole moments. Therefore, it can be concluded that the bulk of carotenoid molecules are aligned parallel to the carotenosome baseplate.

The CD spectrum in the BChl *a* Q_y and Soret region has characteristic shape for exciton interactions. In the BChl Q_y region, the negative CD band (at around 810 nm)

is observed also in the ACD spectrum (with 2-fold increase in magnitude compared to the isotropic CD), while the positive 790 nm CD band is completely suppressed. In the Soret region, the 378 nm band is enhanced in the ACD spectrum, while the 442 nm band is suppressed. A small shoulder is observed around 820 nm in both the CD and ACD spectra; however, we cannot rule out the possibility that it is a purely technical artefact due to a strong xenon-arc line at 826 nm.

An approximate, qualitative interpretation of the ACD spectrum can be done in terms of total exciton transition dipole moments. Considering only the lowest-energy electronic transitions of two interacting molecules, the exciton dimer has two one-electron transitions with orthogonal electric transition dipole moments, giving rise to two bands in the isotropic CD spectrum – at 790 nm and 810 nm. When measuring ACD of the oriented system, transitions parallel to the light wave vector are suppressed (since the scalar product of the light electric field and the electric transition dipole moment component vector is zero). According to these considerations the CD and ACD spectra in the BChl Q_y region can be interpreted as originating from a dimer of BChls with exciton transitions at 790 nm, with transition dipole moment perpendicular to the membrane plane, and at the 810 nm – with transition dipole moment in the membrane plane. For a more complete quantitative description of the ACD, the calculation is done on the basis of monomeric electric and magnetic dipoles and electric quadrupoles (see Methods and Supplementary Methods).

Supplementary Note 4: Structure validation and statistics

The structure was calculated using a total of 60 non-trivial distance constraints, meaning that all cross-peaks present in the DARR spectrum acquired with the short mixing time (20 ms) were not used and all constraints with an assignment possibility corresponding to a distance spanning of one or two bonds only was not used (see Table 1 main text). Out of these 60 constraints 9 were long-range meaning that the smallest residue difference, D, among all assignment possibilities for the restraints were 5 or more or that this was inter-chain constraint. In addition, there was 22 medium range; $1 < D \leq 5$, 26 sequential and 3 intra-residue constraints (most constraints were removed since only spanning one or two bonds). The constraints were implemented as ambiguous upper bound distance restraint with a threshold of

9.0 Å, the restraints have 5.90 assignment possibilities on average. Furthermore, 88 dihedral angle restraints derived using TALOS+ from the assigned chemical shifts were used to restrain the backbone conformation. The structure had 0 violations > 0.5 Å. The structure calculation revealed favourable statistics with angle rms (from ideal angles) = 1.29 degrees, bond rms = 0.011 Å and dihedral angle rms = 1.091 degrees. Processing of the CsmA baseplate structure by the structure validation server, PROCHECK[22] revealed that 85.0%, 11.7%, 2.2%, and 1.1% of the backbone dihedral angles are in the most favoured, additional allowed, generously allowed, and disallowed regions of the Ramachandran plot, respectively. Furthermore, PROCHECK analysis indicated that the side chain dihedral angles were in more favourable conformations on average compared to a typical X-ray structure at 2.0 Å resolution. Comparison of the 10 lowest energy refined structures from the same starting model leads to a heavy atom coordinate rmsd of 1.41 Å for residues 6-48 and Bchl *a* (see Supplementary Fig. 3c and Methods).

The distances measured in the final structure were compared to restraint assignments. An assignment possibility can be both interpreted as intra-chain as well as inter-chain. Some inter-chain combinations are related by symmetry whereas others are unique. For each assignment possibility the observed distance, r_{obs}, in the final structure was evaluated as

$$r_{obs} = \left(\sum_i r_i^{-6}\right)^{-1/6}$$

summing over all non-equivalent unique distances r_i. Due to the symmetry of the oligomeric structure containing one two-fold rotation axis and two different translations there are 38 such different symmetry-unique distances within the 14 chains represented in the structure calculations including the intra-chain distance. The total effective distance, r_{eff}, containing contributions from all observed distances, $r_{obs,j}$, corresponding to all the assignment possibilities, we define as:

$$r_{eff} = \left(\sum_j r_{obs,j}^{-6}\right)^{-1/6}$$

Following this definition, the effective distance is 5.79 Å on average for the restraints and the 90th-percentile is 8.89 Å. For 13 of the restraints, the assignment corresponding to the smallest effective distance was an inter-chain assignment. For completeness, the full list of distance constraints together with all assignment

possibilities and their corresponding effective distance in the final structure is shown in Supplementary Table 1.

As an additional NMR structure validation, back-calculation of CD spectra based on the structure and comparison with the experimental spectrum was used to filter out incorrect structures (see Supplementary Methods section for details). The CD spectrum was calculated for the final refined NMR structure, which showed good agreement with the experimental spectrum (rms = 0.0440 after normalizing spectrum to a maximum absolute value of 1, see Fig 3e) both in terms of sign and g-factor and position of the band at ca. 800 nm. To get the best correlation between calculated and experimental CD spectra, especially in the high energy side of the CD bands at 77 K, vibrations were taken into account in calculations. These vibrations did not have noticeable effect on magnitudes of calculated g-factors, but the high energy side of CD spectrum was well reproduced. The CD spectrum was also calculated at 295 K and compared with observed spectrum (rms = 0.131, see Fig. 3f). There is agreement between the overall shapes of the spectra. A vibration fine structure was not included in this calculation, because we chose to focus on the more sensitive conditions at 77 K, and therefore the observed discrepancy in the intensity between the experimental and calculated spectra on the blue side of the CD band is expected (Fig. 3f main text). Similarly, the observed ACD spectrum was also compared to the structure-based back-calculated showing good agreement with an rms of 0.0844 (see Fig. 3f).
The best of the different structure models were refined against the cryo-EM data. Inspection of the overlays reveals a significantly better fit against the density model (see Supplementary Figs. 6 and 7). In addition, the final structure had a significantly better force field and constraint (NMR + cryo-EM) energy compared to the next best candidates (see Supplementary Fig. 3a). The correlation coefficient between the structure and density model, as calculated by Xplor-NIH using the probDistPot potential[1], for the final structure was 0.192 as compared to 0.149, 0.134, 0.153, 0.028, 0.130, 0.176 for models a-f, respectively, in Supplementary Figure 7. Note that a high value indicates a better agreement, and that the values are relatively low because the 14 peptide chains selected to represent the carotenosome is structure is smaller than the size of the cryo-EM density map.

Supplementary Methods

Structure calculation using a Genetic Algorithm in Symmetry Constraints Space (GASyCS)

The calculation of atomic structures with imposed rotational and translational symmetries can be performed in software packages such as Xplor-NIH using a symmetry potential to equal pairs of distances[23]. In the first phase of the structure calculation, a set of initial monomer structures were calculated using Xplor-NIH and constraints for the backbone dihedral angles inferred from the chemical shifts using TALOS+[7]. However, for the baseplate calculation, these methods had problems generating meaningful models, even with long simulated annealing times. We speculate that the reason for this problem is the high-energy barriers for conversion between two symmetric conformations obeying the symmetry-induced constraints, as these constraints would be violated during the conversion.

We did not find software that addresses these problems specifically suitable for the specific symmetries of the CsmA baseplate[24]. Therefore, we developed new software, GASyCS, for calculating the structure within the space of the eight degrees of freedom for the symmetry (see below). GASyCS has the advantage over existing methods to switch instantaneously between different symmetric conformations and to run many calculations in parallel efficiently. The optimization follows a basin hopping strategy[25], which allows for instant large variations of the protein conformation, using a genetic algorithm[26,27] and subsequent local conformational optimization.

The CmsA baseplate geometry has eight degrees of freedom (see Fig. 2b in main text) for any given choice of monomeric structure. When defining a reference coordinate system with respect to one of the monomers these degrees of freedom are four for dimer symmetry (see Supplementary Note 2 for reasoning for the dimer repeating unit): two for the rotation axis (unit length vector) and two for the position of the rotation axis (the intersection with xy-plane). The four remaining degrees of freedom are two for each of the translations in two directions to define the baseplate array. The two directions must be perpendicular to the dimer rotation axis in order not to break the symmetry of the dimer. These eight parameters are varied in GASyCS.

The geometry of the baseplate is quickly varied using a genetic algorithm framework. In this formalism, the baseplate geometry is parameterized by 8 *genes*, s_1, s_2, …, s_8, encoding the symmetry, which all can take numerical values and one gene for each backbone conformation, b_1, b_2, …, b_N and for each side chain conformation, c_1, c_2, …, c_N, where N is the number of residues (59 here). In addition, there are three more genes for encoding the conformation of the BChl *a* ligand (see below). These backbone and side chain conformations can adopt a limited number of local conformations (here 64). The procedure is initialized by deriving a set of monomer structures (the genetic library) (see above).

As with other genetic algorithms, the key operations in GASyCS are selection, recombination, mutation, evolution and competition (see Supplementary Fig. 10). Firstly, after initialization of the initial population, one or a pair of individuals are selected for rounds of mutation or recombination, respectively, based on the fitness of the individuals. The fitness in this context is the hybrid force field and distance constraint energy (defined below). A new individual (new symmetric conformation) is generated by recombination, i.e., mixing the genes of two conformations or by mutation changing a few genes for one. In GASyCS through evolution, the genes are changed gradually along an energy gradient to optimize the fitness of the individual (optimize the structure). Finally, through competition, the fittest individuals survive into the next generation - and this cycle is repeated a number of times.

In GASyCS, the selection is performed using a so-called normalvariate-rank selection scheme, where the individuals are ranked according to their fitness (total energy) and a random number is drawn from a normal distribution $N(0,\sigma)$. The absolute value of this number is rounded to the nearest integer and the member from the population is chosen as the one with this particular rank. By using smaller standard deviations, σ, fitter individuals will be chosen more frequently, and in GASyCS, this number is decreased for each generation.

Recombination is performed in two different ways. In symmetry recombination, the 8 genes, s_1, s_2, …, s_8, which encode the symmetry are combined producing an offspring having a subset of the 8 genes from the first parent and the remaining from the second parent – e.g., four from each (see Supplementary Fig. 10b). In backbone recombination a so-called double-crossover operation is performed keeping all genes from one parent but replacing a window in the middle of the sequence of the genes

encoding the side chain and backbone conformation with the values from the other parent (see Supplementary Fig. 10c). In this operation, the BChl *a* is considered as a part of the H25 side chain, i.e. whenever H25 is changed the BChl *a* conformation is changed along with it. Mutation is performed by keeping the symmetry and backbone conformation untouched and changing one gene for the side chain conformation, i.e., modifying only the side chain conformation for one single residue (Supplementary Fig. 10d). And again, the BChl *a* conformation is changed together with the H25 side chain conformation. In addition, there are two extra possibilities for mutation: either change only the conformation of the BChl *a* molecule or only the conformation of the phytyl tail of BChl *a*.

In the *evolution* step, the values for the genes encoding the symmetry are systematically varied. This is accomplished using a simulated annealing scheme[28] and/or through simplex minimization in the eight-dimensional space[29]. By rapidly changing geometry and subsequently optimizing it locally, the algorithm can be considered as basin hopping.

If a new individual from either a mutation or a recombination event has lower energy than its predecessor(s) according to a soft Boltzmann criterion, this individual survives in the new generation. Competition between individuals is implemented with two different schemes. In a steady-state scheme, the new fitter individual immediately replaces its weakest parent. In a growth-extinct scheme, the individual is appended to the population and when the population has reached a size threshold individuals are removed from the population (extinction) in a way that both rewards fitness of the individuals but also diversity within the full population (see below).

The fitness of an individual is the total energy E_{tot}, (the lower energy, the better fit), which is the sum of the force field energy, E_{FF}, and experimental constraints energy, $E_{exp} = E_{NMR} + E_{CD} + E_{cryoEM}$, for a given structure. In GASyCS, a pre-optimized local structure for the monomer is provided in the initialization phase and therefore the force field contains no bonded terms but only a Van der Waals repulsion term, E_{VDW}, similar to other software. I.e., if the distance, d, between two atoms is below a certain threshold, t, the VDW term is the square of the difference $E_{VDW} = (t-d)^2$. The precise definition of the VDW term depends on the representation of the molecular geometry (coarse grain, heavy or full atoms, which can be different for each generation, see below). E_{NMR} is a sum of two terms; an ambiguous distance constraints term

implemented as in Xplor-NIH with a soft-square shape of the potential and, in the final stages of the calculations, a chemical shift pseudo-energy derived using shAIC[8] based on the agreement between observed and back-calculated chemical shift. The constraints from CD, E_{CD}, is as sum of terms for orientation and distance based on earlier observation[30]. The orientation term enforces orthonormality between the baseplate and porphyrin ring normals (see Supplementary Note 2) requiring an angle of at least 70° using an expression similar to the E_{VDW} term. The distance term constraints the distance between the closest Mg-Mg pairs to be between 15 and 25 Å (to account for the small observed g-factor) using a square well quadratic potential as with classical distance constraints derived from NMR. Based on cryo-EM observations, the E_{cryoEM} term enforces that at least one of the translations between the monomers has a length of 33 Å ± 5 Å, which is implemented as above with a square well quadratic potential.

The genetic algorithm starts with an initialization of the geometry and then runs in three stages with a number of generations in each and 19 generations in total (see details in Supplementary Table 4). The phases can be classified by the representation of the atomic structure used for defining E_{VDW}. In the first phase, the protein structure have a coarse grain representation, next phase all heavy atoms, and in the final phase, full-atom representation. The genetic operations and competition modes and several other parameters are different in the different phases; all the important details are shown in Supplementary Information Table 1. In the final phase, a chemical shift pseudo energy calculated by shAIC is included in the expression for E_{NMR}. Following this scheme, 8 structures were produced after the 19 generations and this procedure was parallelized on 16 processors producing 128 structures in total. The software, GASyCS, is available upon request to the corresponding authors.

Structure calculation using GASyCS. Defintion of the extinction evaluation

After each generation the individuals compete and through extinction it is evaluated based on the extinction energy which subgroup of the population survived to the next generation. The combined energy, E_{comb}, is a sum of the energy (the fitness) for the individual, E_{tot}, as described in additional Methods and the diversity reward, ρ,

$$E_{comb} = E_{tot} + w_{div}\rho$$

where w_{div} is the weight for the diversity. The diversity reward, ρ, is calculated based on the pairwise "distance", d_{ij}, between two individuals, which is a measure of the similarity between two structures. The distance is a sum, from three contributions:

$$d_{ij} = d_{ij}^{s} + d_{ij}^{b} + d_{ij}^{c}$$

The distances are related to symmetry, backbone and side-chain geometry, respectively. The former is defined as:

$$d_{ij}^{s} = \log(D_{ij}^{s})$$

where

$$D_{ij}^{s} = \sqrt{\frac{1}{8}\sum_{n=1}^{8} w_n^s \left(s_n^i - s_n^j\right)^2}$$

where s_n^k is the nth gene encoding for symmetry for the kth individual and w_n^s is the symmetry weight for the nth gene, which were set to 1 for genes encoding distances and 49.0 for genes encoding angles (spherical coordinates).

Furthermore, the distance related to the backbone geometry is set to 0.0 if the backbone geometries are identically and else defined as:

$$d_{ij}^{b} = 50.0 + 10.0 \times \log(1 + D_{ij}^{b})$$

where

$$D_{ij}^{b} = \sum_{n=1}^{N} \delta(b_n^i, b_n^j)$$

where the sum runs from 1 to 59 in this case and here δ is the Dirac delta function comparing two genes, b_n^i, b_n^j, (integers) encoding for the backbone geometry,

$$\delta(k,m) = \delta_{km} = \begin{cases} 1 \; if \; k = m \\ 0 \; if \; k \neq m \end{cases}$$

Similarly, the distance related to the side-chain geometry is:

$$d_{ij}^{c} = 20.0 + 4.0 \times \log(1 + D_{ij}^{c})$$

where

$$D_{ij}^{c} = \sum_{n=1}^{N} \delta(c_n^i, c_n^j)$$

The extinction algorithm, which defines the next generation, is initialized by placing the four individuals with lowest energy, E_{tot}, in the new generation. After the initialization and in subsequent step until the threshold size is reached, the individual with lowest combined energy, E_{comb} (see above), is added to the new generation. The diversity reward for the kth individual, ρ_k, used in the definition of E_{comb} is calculated as:

$$\rho_k = \left(\frac{1}{N}\sum_{i=1}^{N} d_{ik}{}^{-6}\right)^{-1/6}$$

where the sum over i runs across the individuals already present in the new generation.

Image processing of cryo-EM data and 3D reconstruction

A total of 270 CCD frames were selected for processing. Portions of individual baseplates from different orientations with a striated appearance were selected using e2boxer.py in the EMAN2 software package[31,32]. Particles were picked manually to prevent selection of damaged or overlapping particles, resulting in a total of 1750 individual particles. Initial CTF-correction and 2D class-averaging were performed in EMAN2, yielding 48 class averages (Supplementary Fig. 8a). In a second round, 266 weak-contrast particles tentatively representing top-views were selected and processed in the same way.

The classes were subjected to distance analysis in a procedure where power spectra of individual class-averages were calculated using Spider[33] and the strongest Fourier-components were located by a peak-search algorithms. The resolution for the two strongest Friedel pairs were calculated and interpreted as repeat distances (or thickness) in the corresponding projections (Fig. 2d,e,f inserts). Three characteristic 2D class averages (Fig. 2d,e,f main text), which appear as a line of equidistant "beads" (beady view), a single stripe, and a set of aligned stripes, respectively were initially identified. Repeat distances for the beady view from different class-averages were very well conserved at 33 Å (in correspondance with previously reported results[10,34,35]), whereas the thickness of the beads and stripes were in the range of 41-47 Å.

By including the minimal set of three class-averages a one-layered reconstruction was obtained, in accordance with the expected shape of the baseplate (Supplementary Fig. 8b). Comparison between class averages and projections from the initial model revealed a very good match between the beady view and single stripe view (Main text, Figs. 2 d&e, g&h, whereas the multiple striped view showed a wider spacing than the projection direction perpendicular to the baseplate plane.

To obtain a refined reconstruction, defocus values were determined using ctffind3[36] and found to be in the range 1.7-4.0 µm. Subsequent ctf-correction was performed by

phase-flipping in Spider[33]. To obtain a refined 3D reconstruction, the 1790 individual particles were initially subjected to reference free inplane-alignment with Sparx[37] in order to establish a common origin and subsequently in Euler angle space using Strul[38] with the initial model as a starting model. A new 3D model was calculated using weighted back-projection in Spider. The initial and refined reconstruction models are shown overlaid in Supplementary Fig. 8b

Refinement of individual baseplate images

In order to increase the resolution of the reconstruction, the set of individual images were aligned in 3D, as described above (see Methods). Alignment of individual particles versus a non-isotropic reference volume requires a strategy about the handling of reference projections. In this case, the reconstruction with the most regular lattice shape was obtained when the top-view was omitted from the reference-projections, using only the two projections in the plane of the baseplate as described above. To vindicate this procedure, we note that if both the beady view and the striped view (see Fig. 2d,e) would have displayed repeating distances along the stripes, a reliable top-view would have been essential to lock the first two views relative to each other. In our case however, the striped view essentially contains information about the thickness of the baseplate. Thus in the alignment of individual particles, the striped view will mainly tend to constrain the density of the reconstruction to the XY-plane with rotational freedom around the Z-axis (justified by our prior assumption about the topology of the baseplate) whereas the better resolved beady view will determine the orientation of particles in the XY-plane/plane of the baseplate. The mesh like structure of the final cryo-EM reconstruction raises the question whether the observed features in the refined reconstruction could be the consequence of a 90º juxtaposition of orientations. However, examination of the projections (examples shown in Fig. 2g-i) of the reconstruction reveals the clear differences between these two projection directions. To evaluate the hypothesis that a small proportion of the images would align at 90º to their correct orientation (possibly due to translational misalignment, thus giving rise to the observed criss-cross pattern), the half of particles with the lowest correlation in the 3D-alignment were excluded from the reconstruction procedure. The resulting map revealed a similar chequered pattern, but with a noisier appearance. The same observation is made when the top-view is included in the reference projection set (data not shown).

Further analysis of the data reveal the presence of low-contrast class averages with barely distinguishable mesh-like pattern with power-spectra (Fourier transforms) with two repeat distances at 33.8 and 35.7 Å respectively and at an angle of 81°, consistent with the top views of the initial and refined 3D reconstructions (Fig. 2c,f).

A superposition of the refined and the initial reconstructions show them to be similar, the main differences being that the small bulges visible in the initial reconstruction become bridging features in the refined reconstruction (Supplementary Fig. 8b). While these bulges suggest a pattern for protein-protein contacts, the same molecular conformation comes out as the most favourable irrespective of which EM-map is used for spatial constraints (Supplementary Fig. 3b, data for fit against initial model not shown), suggesting that the structural information in the beady view is what governs this fitting. The resolution of the final reconstruction is 25.5 Å (19.1 Å), according to the FSC=0.5 (FSC=0.143) criteria (Supplementary Fig. 3b). This FSC-assessment is performed by dividing the data into two random sets, aligning once against the initial model and performing 3D reconstruction as described above, thus no successive accumulation and over-fitting against spurious noise in the raw data as required by a gold-standard resolution estimate.

CD spectroscopy and back-calculation of CD-spectra by quantum chemical methods

Initially we filter out incorrect baseplate model structures by using exciton theory to reproduce room temperature absorption and (isotropic) CD spectra of baseplate in the Q_y spectral region[30,39]. In our calculations, the model structures consider ten side-by-side oriented rod-like CsmA arrays with twenty BChl *a* pigments per array. Each pigment contains electronic ground state and the first singlet excited electronic Q_y state). In exciton Hamiltonian, monomeric transition energy (site energy) was 12530 cm^{-1} (798 nm) and absolute value of the Q_y transition dipole vector was taken as 6.13 D pointing in the direction of N(A)-N(C) atoms of BChl *a* (according to IUPAC numbering scheme of BChl). Homogeneous line width of 180 cm^{-1} and Gaussian random distribution of electronic transition energies (220 cm^{-1}) and exciton couplings (10% of the value of the coupling strength) was used to generate inhomogeneous broadening of spectral line shapes of Q_y band at 295 K. All structures with wrong sign of the CD band at 800 nm, too weak CD/Absorbance intensity ratio (g-factor) at 800

nm or wrong absorption band shape as compared to the experimental one were filtered out.

To validate the final structure, we back-calculated 77K CD spectrum of the CsmA baseplate. Exciton theory with calculated normal mode vibrations and calculated Franck-Condon (FC) factor was used[40]. The structures used for computation consist of five side-by-side oriented rod-like CsmA arrays with ten BChl *a* pigments per array. Each pigment contains electronic ground state and the first singlet excited electronic Q_y state. Both states contain vibration ground state, fundamental modes of vibrations, ten overtones of modes and combinations of modes. It was assumed that the system will undergo a vertical FC transition and Boltzmann distribution was used to generate initial thermal occupations of vibration states at 77 K. Semiempirical PM6 method[41] with full configuration interaction approaches was used to optimized geometries of the electronic states (needed to calculate normal modes of vibrations), to calculate normal modes of the electronic states and to calculate an electronic transition dipole vector between the electronic states of pigments[42]. The four highest occupied and four lowest unoccupied molecular orbitals were used to form the active CI space of pigment in the calculations. The FC factors were evaluated by calculating overlap integrals between the vibration eigenstates in their respective electron state using harmonic oscillator wave functions. The FC factors modulate magnitude of electrical transition dipole vectors, and thus exciton coupling strengths between the pigments of the baseplate. Vibronic transitions from the thermally occupied states to the all final states of the singlet excited electronic state whose FC factor is larger than 0.1 * the mean value of calculated coefficients and transition energy is below 14900 cm^{-1} (670 nm) were used in exciton calculations. In exciton Hamiltonian, monomeric transition energy (site energy) was 12530 cm^{-1} (798 nm, for 0-0 transition) and absolute value of the Q_y transition dipole vector was taken as 6.13 D. Gaussian random distribution of electronic transition energies (180 cm^{-1}) and exciton couplings (10% of the value of the coupling strength) was used to generate inhomogeneous broadening of spectral line shapes of Q_y band. Homogeneous line width (full width at half maximum) of 4 cm^{-1} was used for all vibronic bands.

Back-calculation of anisotropic CD-spectra by quantum chemical methods

In anisotropic CD (ACD) calculations we used exactly the same exciton Hamitonian as in the ordinary CD calculations at room temperature (see above). The only

difference between ACD and CD calculations was that in the ACD calculations the baseplate structure was spatially oriented, having similar spatial orientation relative to the propagation of the measuring beam as carotenosomes in our experimental gel-squeezing ACD measurement. The ACD technique provides additional information on the architecture of BChl a pigments of the baseplate in the macroscopic structure of the carotenosome. The propagation of light was parallel to the baseplate normal in our studies. Directional anisotropic CD intensity was calculated by using equations (ACD 1 - ACD 5)[39,40,43,44]: equation (ACD2) shows the electric dipole-electric dipole ACD contributions. Electric dipole-magnetic dipole and electric dipole-electric quadrupole contributions for ACD are shown in eqs. (ACD3)-(ACD5).

$$ACD[\vec{u},\omega] = \sum_s ACD_s[\vec{u},\omega] = \sum_s (CD_s^1[\vec{u},\omega] + CD_s^2[\vec{u},\omega])$$

(ACD 1)

where

$$CD_s^1[\vec{u},\omega] = -m^2\omega_s^2 Im\left\{\sum_{k,l} c_{sk}c_{sl}^* e^{-i\frac{\omega}{c}\vec{u}\cdot\vec{R}_{kl}}[\vec{u}\cdot\vec{\mu}_k \times \vec{\mu}_l]\right\},$$

(ACD 2)

$$CD_s^2[\vec{u},\omega] = \frac{\omega}{c} Re\left\{\sum_{k,l} c_{sk}c_{sl}^* e^{-i\frac{\omega}{c}\vec{u}\cdot\vec{R}_{kl}}[\vec{u}\cdot(\boldsymbol{M}_{skl} + \boldsymbol{Q}_{skl})\cdot\vec{u}]\right\},$$

(ACD 3)

$$\boldsymbol{M}_{skl} = m^2\omega_s(Im\{\vec{\mu}_k\cdot\vec{m}_l\boldsymbol{I} - \vec{\mu}_k{:}\vec{m}_l\} + Im\{\vec{\mu}_l\cdot\vec{m}_k\boldsymbol{I} - \vec{\mu}_l{:}\vec{m}_k\}) \text{ , and}$$

(ACD 4)

$$\boldsymbol{Q}_{skl} = \frac{1}{2}m^2\omega_s^2\{\boldsymbol{q}_k \times \vec{\mu}_l + \boldsymbol{q}_l \times \vec{\mu}_k\}.$$

(ACD 5)

In the equations, m is mass of an electron, ω is an angular frequency, c_{sk} is the k:th element of the eigenvector for the sth exciton state, c is a speed of light, $\vec{u}$ is unit vector in the direction of light propagation, $\vec{R}_{kl}$ is the position vector between molecules k and l, $\vec{\mu}_k$ is the electronic transition dipole moment vector in molecule k, $\vec{m}_k$ is the magnetic transition dipole vector in molecule k, $\boldsymbol{q}_k$ is the electronic

quadrupole transition moment in molecule *k*, ***I*** is a unit tensor and the symbol **:** denotes a dyadic vector product.

In the ACD calculations, the Q_y electronic transition dipole moment vector, the Q_y magnetic transition dipole vector and the Q_y electric quadrupole transition moment are identical for the individual BChl *a* molecule, except for the rotations following the orientation of the BChls *a* of the baseplate. These vectors and quadrupoles were calculated by using time-dependent density-functional CAM-B3LYP/6-311G(d,p) method. CAM-B3LYP/6-311G(d,p) optimized BChl *a* – pyridine ligand complex, in which one pyridine ligands to the central Mg atom, was used as a structural model for the histidine-bound five-coordinated BChl *a* molecule in a real CsmA protein structure.

Supplementary References

1 Gong, Z., Schwieters, C. D. & Tang, C. Conjoined Use of EM and NMR in RNA Structure Refinement. *PLoS ONE* **10** (2015).

2 Khor, C. C. *et al.* Genome-wide association study identifies FCGR2A as a susceptibility locus for Kawasaki disease. *Nature Genetics* **43**, 1241-1246 (2011).

3 Nielsen, J. T. *et al.* Unique Identification of Supramolecular Structures in Amyloid Fibrils by Solid-State NMR Spectroscopy. *Angew. Chem. Intl. Edit.* **48**, 2118-2121 (2009).

4 Larkin, M. A. *et al.* Clustal W and Clustal X version 2.0. *Bioinformatics* **23**, 2947-2948 (2007).

5 Schwieters, C. D., Kuszewski, J. J., Tjandra, N. & Clore, G. M. The Xplor-NIH NMR molecular structure determination package. *J. Magn. Reson.* **160**, 65-73 (2003).

6 Schwieters, C. D., Kuszewski, J. J. & Clore, G. M. Using Xplor-NIH for NMR molecular structure determination. *Prog. Nucl. Magn. Reson. Spectrosc.* **48**, 47-62 (2006).

7 Shen, Y., Delaglio, F., Cornilescu, G. & Bax, A. TALOS plus : a hybrid method for predicting protein backbone torsion angles from NMR chemical shifts. *J. Biomol. NMR* **44**, 213-223 (2009).

8 Nielsen, J. T., Eghbalnia, H. R. & Nielsen, N. C. Chemical shift prediction for protein structure calculation and quality assessment using an optimally parameterized force field. *Progr. Nucl. Magn. Reson. Spectrosc.* **60**, 1-28 (2012).

9 Pettersen, E. F. *et al.* UCSF Chimera--a visualization system for exploratory research and analysis. *J. Comput. Chem.* **25**, 1605-1612 (2004).

10 Oostergetel, G. T., van Amerongen, H. & Boekema, E. J. The chlorosome: a prototype for efficient light harvesting in photosynthesis. *Photosynth. Res.* **104**, 245-255 (2010).

11 Staehelin, L. A., Golecki, J. R. & Drews, G. Supramolecular organization of chlorosomes (chlorobium vesicles) and of their membrane attachment sites in Chlorobium limicola. *Biochim Biophys Acta* **589**, 30-45 (1980).

12 Psencik, J. *et al.* Structure of chlorosomes from the green filamentous bacterium Chloroflexus aurantiacus. *J. Bacteriol.* **191**, 6701-6708 (2009).

13 Olson, J. M. Chlorophyll organization in green photosynthetic bacteria. *Biochim Biophys Acta* **594**, 33-51 (1980).

14 Kulminskaya, N. V. *et al.* In situ solid-state NMR spectroscopy of protein in heterogeneous membranes: the baseplate antenna complex of Chlorobaculum tepidum. *Angew. Chem. Intl. Ed. Engl.* **51**, 6891-6895 (2012).

15 Alia, A., Buda, F., de Groot, H. J. & Matysik, J. Solid-state NMR of nanomachines involved in photosynthetic energy conversion. *Ann. rev. biophys.* **42**, 675-699 (2013).
16 Alia *et al.* Ultrahigh field MAS NMR dipolar correlation spectroscopy of the histidine residues in light-harvesting complex II from photosynthetic bacteria reveals partial internal charge transfer in the B850/His complex. *J. Am. Chem. Soc.* **123**, 4803-4809 (2001).
17 Alia *et al.* Heteronuclear 2D (H-1-C-13) MAS NMR resolves the electronic structure of coordinated histidines in light-harvesting complex II: Assessment of charge transfer and electronic delocalization effect. *J. Biomol. NMR* **28**, 157-164 (2004).
18 Barraud, P., Schubert, M. & Allain, F. H. A strong 13C chemical shift signature provides the coordination mode of histidines in zinc-binding proteins. *J. Biomol. NMR* **53**, 93-101 (2012).
19 Baker, B. R. & Garrell, R. L. g-Factor analysis of protein secondary structure in solutions and thin films. *Faraday Discuss.* **126**, 209-222 (2004).
20 Shibata, Y., Saga, Y., Tamiaki, H. & Itoh, S. Anisotropic distribution of emitting transition dipoles in chlorosome from Chlorobium tepidum: fluorescence polarization anisotropy study of single chlorosomes. *Photosynth. Res.* **100**, 67-78 (2009).
21 Kell, A., Chen, J., Jassas, M., Tang, J. K. & Jankowiak, R. Alternative Excitonic Structure in the Baseplate (BChl a-CsmA Complex) of the Chlorosome from Chlorobaculum tepidum. *J. Phys. Chem. Lett* **6**, 2702-2707 (2015).
22 Laskowski, R. A., Macarthur, M. W., Moss, D. S. & Thornton, J. M. PROCHECK - a program to check the stereochemical quality of protein structures. *J. Appl. Crystall.* **26**, 283-291 (1993).
23 Nilges, M. & Brunger, A. T. Successful prediction of the coiled coil geometry of the GCN4 leucine zipper domain by simulated annealing: comparison to the X-ray structure. *Proteins* **15**, 133-146 (1993).
24 Bardiaux, B., van Rossum, B. J., Nilges, M. & Oschkinat, H. Efficient modeling of symmetric protein aggregates from NMR data. *Angew. Chem. Intl. Ed. Engl.* **51**, 6916-6919 (2012).
25 Wales, D. J. & Doye, J. P. K. Global optimization by basin-hopping and the lowest energy structures of Lennard-Jones clusters containing up to 110 atoms. *J. Phys. Chem. A* **101**, 5111-5116 (1997).
26 Mitchell, M. *An introduction to Genetic Algorithms.* (MIT, 1999).
27 Michalewicz, Z. *Genetic Algorithms + Data Structures = Evolution Programs.* (Springer, 1996).
28 Kirkpatrick, S., Gelatt, C. D., Jr. & Vecchi, M. P. Optimization by simulated annealing. *Science* **220**, 671-680 (1983).
29 Nelder, J. A. & Mead, R. A Simplex-Method for Function Minimization. *Comput. J.* **7**, 308-313 (1965).
30 Pearlstein, R. M. in *in "Chlorophylls"* (ed H. Scheer) 1047-1078 (CRC Press, Boca Raton, 1991).
31 Tang, G. *et al.* EMAN2: an extensible image processing suite for electron microscopy. *J. struct. biol.* **157**, 38-46 (2007).
32 Ludtke, S. J., Baldwin, P. R. & Chiu, W. EMAN: semiautomated software for high-resolution single-particle reconstructions. *J. struct. biol.* **128**, 82-97 (1999).
33 Shaikh, T. R. *et al.* SPIDER image processing for single-particle reconstruction of biological macromolecules from electron micrographs. *Nature protocols* **3**, 1941-1974 (2008).
34 Psencik, J. *et al.* Structural and functional roles of carotenoids in chlorosomes. *J. Bacteriol.* **195**, 1727-1734 (2013).
35 Oostergetel, G. T. *et al.* Long-range organization of bacteriochlorophyll in chlorosomes of Chlorobium tepidum investigated by cryo-electron microscopy. *FEBS Lett.* **581**, 5435-5439 (2007).
36 Mindell, J. A. & Grigorieff, N. Accurate determination of local defocus and specimen tilt in electron microscopy. *J. struct. biol.* **142**, 334-347 (2003).
37 Hohn, M. *et al.* SPARX, a new environment for Cryo-EM image processing. *J. struct. biol.* **157**, 47-55 (2007).
38 Lindahl, M. Strul--a method for 3D alignment of single-particle projections based on common line correlation in Fourier space. *Ultramicroscopy* **87**, 165-175 (2001).
39 Davydov, A. S. *Theory of molecular excitons.* (McGraw-Hill, 1962).
40 Linnanto, J. M. & Korppi-Tommola, J. E. I. Modelling excitonic energy transfer in the photosynthetic unit of purple bacteria. *Chem. Phys.* **357**, 171-180 (2009).
41 Stewart, J. J. P. Optimization of parameters for semiempirical methods V: Modification of NDDO approximations and application to 70 elements. *J. Mol. Model.* **13**, 1173-1213 (2007).

42 Ratsep, M., Linnanto, J. & Freiberg, A. Mirror symmetry and vibrational structure in optical spectra of chlorophyll a. *The J. chem. phys.* **130**, 194501 (2009).

43 Hansen, A. E. B., K. L. Ab initio calculations and display of enantiomeric and nonenantiomeric anisotropic circular dichroism: The lowest pi ->pi* excitation in butadiene, cyclohexadiene, and methyl-substituted cyclohexadienes. *J. Phys. Chem.* **104**, 11362-11370 (2000).

44 Hansen, A. E. Molecular exciton approach to anisotropic absorption and circular dichroism I. General formulation. *Monatsh. Chem.* **136**, 253-273 (2005).
